# Interperformer coordination in piano-singing duo performances: phrase structure and empathy impact

**DOI:** 10.1007/s00426-023-01818-8

**Published:** 2023-04-19

**Authors:** Sara D’Amario, Harald Schmidbauer, Angi Roesch, Werner Goebl, Anna Maria Niemand, Laura Bishop

**Affiliations:** 1 Department of Music Acoustics-Wiener Klangstil, mdw–University of Music and Performing Arts Vienna, Anton-von-Webern-Platz 1, 1030 Vienna, Austria; 2 Shanxi University of Finance and Economics, Taiyuan, Shanxi China; 3 FOM University of Applied Sciences, Munich, Germany; 4 RITMO Centre for Interdisciplinary Studies in Rhythm, Time and Motion, University of Oslo, Oslo, Norway; 5 Department of Musicology, University of Oslo, Oslo, Norway

## Abstract

Musicians’ body motion plays a fundamental role in ensemble playing, by supporting sound production, communication, and expressivity. This research investigates how Western classical musicians’ head motion during ensemble performances relates to a piece’s phrase structure and musicians’ empathic perspective taking (EPT) profile. Twenty-four advanced piano and singing students took part in the study, and their EPT score was pre-assessed using the Interpersonal Reactivity Index. High and low EPT duos were formed, and musicians were paired with a co-performer from the same and the other EPT group. Musicians rehearsed Fauré’s Automne and Schumann’s Die Kartenlegerin, and performed the pieces one time before and three times after rehearsal. Motion capture data of the musicians’ front head, audio, and MIDI recordings of the performances were collected and analysed. Similarity in musicians’ head motion and tendency to lead/lag their co-performer were computed by extracting, respectively, power and phase difference of the cross-wavelet transforms of the velocity curves of each paired marker. Results demonstrate that the power of interperformer coordination corresponds to the piece’s phrase levels and that singer’s EPT can impact the leader-follower relationships between musicians, depending on piece and take number. In the Fauré piece, the higher the singer’s EPT score, the higher the tendency for the singer to lead and pianist to follow in take 3, and the lower the tendency for the singer to lead and pianist to follow in take 2. These results contribute to a further understanding of the mechanisms underpinning social interactions, by revealing the complexity of the association between empathy and body motion in ensembles in promoting and diffusing leadership between musicians.

**Supplementary Information:**

The online version contains supplementary material available at 10.1007/s00426-023-01818-8.

## Introduction

Music ensemble playing is a peculiar, complex and naturalistic form of nonverbal joint action that is of scientific interest to researchers in music psychology, performance science and neuroscience (Keller et al., 2014). In order to achieve a cohesive and expressive performance, trained ensemble musicians in the Western classical tradition coordinate and adjust their tempo, sound and bodily gestures to align with that of their co-performer(s), agree on a shared understanding of the composer’s intentions, predict co-performer(s) individual intentions, and often monitor audience responses.

Musicians’ body motion during ensemble performance is continuous and multifunctional. Some aspects of motion are directly involved in sound production; other aspects, often referred to as “ancillary motion”, support sound-producing motion, facilitate interpersonal communication and interactions, help with achieving expressive goals, and provide visual expressive cues to audience/co-performers (Jensenius et al., 2010). Ancillary motion relates to the performer’s understanding of the piece’s structural significance, to the coordination of musical phrases (Thompson & Luck, 2011), and to expressive intentions (Dahl & Friberg, 2007; Thompson & Luck, 2011). In sum, body motion can help co-performers to achieve their intended musical interpretation and intentions and ease coordination.

In this study, we analysed interperformer coordination, as manifested in musicians’ continuous head motion during duo performances, in relation to the empathic perspective taking ability of the musicians and the structure of the music performed.

In the following sections, we discuss the current understanding of the role of body motion in ensemble performance and the relationships between interperformer coordination and empathy, review methodologies for the analysis of musicians’ bodily coordination, and then pose hypotheses for the current study.

### Body motion in ensemble performance

Musicians’ body motion plays a fundamental role in ensemble playing. Some studies have shown that head movements can reflect the emotionally expressive intentions that musicians aim to convey. For example, the information flow between members of a professional trio, as measured in their anterior-posterior head sway, was found to be higher when performing with emotional expression than without (i.e., a mechanical performance) (Chang et al., 2019). Pianists’ head movement velocities have also been found to differ depending on the pianists’ expressive intent, and to be higher in expressive serene conditions than in sad, allegro, and overexpressive conditions (Castellano et al., 2008).

Some emotional intentions that musicians aim to convey during solo performances can also be communicated to observers through body motion. In an experiment involving participants’ ratings of silent videos of marimba clips in which a musician was instructed to express different intentions, Dahl and Friberg (2007) found that the intentions of happiness, sadness and anger of marimba performances were successfully communicated by the musician to participants. Anger was mostly represented through jerky movements, happiness was represented through large movements, and sadness was represented through slow and smooth movements.

Musicians’ body motion can also reveal leader-follower relationships between musicians during ensemble playing. Designated leaders in piano duos tend to raise their fingers higher than designated followers (Goebl & Palmer, 2009), and a study of coupling in the body sway of string quartet players shows that assigned leaders influence the others more and are less influenced by others than are followers (Chang et al., 2017). Studies on leadership in string quartets in ecologically more valid situations (i.e., without researchers assigning the role of leader) highlight the complexity and differentiated patterns of dependencies rather than the more traditional role allocation that attributes leadership to the first violin (Glowinski et al., 2012; Timmers et al., 2014). These results demonstrate that musicians’ body movements reflect musical roles of leader or follower, and suggest that the leader-follower relationship can impact the way musicians adapt their behaviours to that of the co-performers during ensemble performance. These findings also imply that leader-follower roles are flexible and may be exchanged back and forth during a piece.

Furthermore, body motion in music ensembles can facilitate interpersonal communication, coordination and interaction. Certain acceleration patterns in leaders’ head gestures, comprising the period of deceleration following acceleration peaks, were found to communicate beat position in piano duos synchronizing piece entrances (Bishop & Goebl, 2017). Also found were increases in interperformer coordination, quantity of head movements and explicit cueing gestures in piano and clarinet duos during irregularly timed passages compared to other parts of a piece, demonstrating a tendency to interact visually during periods of temporal instability (Bishop et al., 2019). Pianists’ head movements in piano duos were found to be more synchronized when auditory feedback was reduced (i.e., both pianists could hear only themselves though playing together), compared to when designated leaders could hear only themselves, whilst followers had full feedback, and also compared to when both pianists had full auditory feedback. These results demonstrate that musicians can adapt their body motion as a way to maintain successful interpersonal coordination if auditory information is incomplete (Goebl & Palmer, 2009).

Taken together, these studies demonstrate that musicians’ body motion can relate to higher-order piece structure, reflect the dynamics of nonverbal information flow and facilitate communication between co-performers during ensemble performances.

### Relationships between interperformer interactions and empathy

Joint action activities can enhance the capacity to understand what another person is experiencing, which is generally referred to as “empathy”, a term coined in 1909 by Edward Titchener as translation from “Einfühlung” (“feeling into”) used in the German aesthetic theory of the nineteenth century (Titchener, 1909). Empirical investigations in joint action activities have recently revealed the bidirectional relationship between interpersonal coordination and empathy, which represents a fundamental social-psychological component of ensemble playing.

A body of research has analysed whether and how interpersonal interactions in joint music making enhance empathy related skills in children and adults. Results demonstrate that long-term participation in group-based, interactive musical activities increases emotional empathy scores in school-aged children (Rabinowitch et al., 2012), and sympathy and pro-social skills in children having poor pro-social skills before the musical intervention took place (Schellenberg et al., 2015). Interestingly, it has been demonstrated that the ensemble experience of college music students sampled in the United States and in South Korea relates to the student’s empathy skills (Cho, 2019; Cho & Han, 2021).

In addition to studies analysing the impact of ensemble playing on empathy, research has also demonstrated the impact of empathy on joint action. Empathy impacts the three core cognitive-motor skills (i.e, anticipation, adaptation, and attention) underpinning interpersonal coordination in expressive ensemble performance (Keller, 2014). In a multidimensional approach to empathy, the Empathic Perspective Taking (EPT) trait,^1^ a component of cognitive empathy referring to the individual tendency to adopt the psychological point of view of other(s) (Davis, 1980, 1983), has been the focus of a number of investigations in ensemble performance.

Cognitive neuroscience studies, analysing joint-action in “simulated” piano duos (i.e., pianists who believe that they are playing along with a second pianist, though performing with a pre-recorded performance), show that higher empathic perspective-taking scores are correlated with higher microtiming adaptation (Novembre et al., 2014). Similar studies in this field have shown that more empathic musicians rely on motor simulation to a higher degree, since EPT was found to be positively correlated with neurophysiological measures (i.e., corticospinal excitability recorded by means of electromyography) indicating pianists’ ability to represent their co-performer’s actions in their own motor system (Novembre et al., 2012). A recent study further investigated the role of EPT during a joint music-making task, by demonstrating that this promotes interpersonal synchronization accuracy measured at low-order note-to-note synchronization, and that designated followers with high EPT scores show greater predictive skills than the low EPT followers, as they lagged behind leaders to a smaller degree (Novembre et al., 2019).

In summary, these results demonstrate that empathy improves synchronization skills. However, these results have not been corroborated in the context of ecologically valid ensemble performances. Empathy may also facilitate coordination at a deep expressive level, leading to effects on unintentional coordination of musicians’ head motion in ensembles. Empathy might also impact leader-follower relationships by promoting followers’ greater abilities in anticipating leaders’ behaviour, due to the enhancement of predictive skills and simulation mechanisms. The goal of our study was to shed some more light on this aspect by seeking evidence of the impact of EPT on interperformer coordination of piano singing-duos, operationalised in terms of musicians’ head motion.

### Interperformer coordination measures

Measures of interpersonal coordination are meant to characterise the synchronicity of two person-related time series of sensor readings. The investigation can be performed in the time- and/or frequency-domain (Issartel et al., 2015). Time-lagged cross-correlations methods may allow for an adequate measurement of the synchrony in applications to event timing data. However, cross-correlation methods are prone to producing spurious results when applied to sensor readings of musicians’ body movements (Dean & Dunsmuir, 2016): in response to the flow of the musical score, the sensor data stream is smoothly changing, hence auto-correlated as well as non-stationary, i.e. with statistical properties changing over time. Frequency-domain analyses of time-series data are of particular interest to studies of ensemble performance, in which ensemble musicians’ expressive movements often reflect the hierarchical structure of the music, defined by subdivisions which are found within beats, within bars, within phrases, within sections, within pieces (Demos & Chaffin, 2017; Demos et al., 2017). A well-known mathematical method for spectrum analysis is the Fourier transform, which computes the power of individual sinusoidal components. This specific method is very efficient with interactions relying on stable frequencies across time, as it assumes the stationarity of the processes in time (Issartel et al., 2006); however, it can present a practical limitation in the case of movement interactions in ensemble performances, where a dominant rhythm cannot always be set and does not readily translate into fixed-frequency body oscillations, as faster and slower bodily oscillations can frequently occur.

An alternative method that circumvents the rigid assumptions of Fourier analysis is wavelet analysis. It allows for variable frequencies across time and can thus capture also intermittent oscillations in the time-frequency domain (Torrence & Compo, 1998) as well as nested rhythmic structures (Schmidt et al., 2012; Washburn et al., 2014). When applied to a single time series, the wavelet transform will provide information about the time-frequency structure of the series; that is, which frequency is important at what time.

The joint wavelet transformation of two time series yields the so-called cross-wavelet transform (CWT), which provides information about which frequency is an important constituent in both series. Thus, applied to time series of two musicians’ sensor readings, CWT analysis gives insight into the strength, or power, of this joint frequency in bodily oscillations. In addition, the oscillations at a joint frequency can be in phase (e.g., both musicians moving forward and backward in sync), or out of phase (e.g., one musician moving forward while the other is moving backward). At any point in time, CWT analysis allows to measure the degree of synchronization of joint-frequency oscillations by means of the phase difference, or phase shift. Therefore, the concept of phase difference will not only indicate whether the two oscillations are in phase or out of phase, but also which oscillation is leading (i.e., reaches its peak or trough first, within a cycle). CWT analysis thus permits the identification of patterns of coordination between two musicians and also provides an indication of the tendency to lead or follow. The CWT has been found useful in a variety of disciplines, including geophysics (Grinsted et al., 2004), electroencephalographic studies (De Carli et al., 2004), and also social psychology measuring interpersonal interactions in dance settings (Washburn et al., 2014) and between co-actors during joke-telling tasks (Schmidt et al., 2014).

A few studies have already demonstrated the potential of using CWT in the context of music ensemble performances. Walton et al. (2015) showed different patterns in the lateral forearms movements of piano players emerging as a function of the musical context, when piano duos improvised and played in synchrony with an ostinato backing track. Specifically, coordination between pianists was stronger when playing in synchrony rather than improvising, and synchronization power was strongest at the four second period length, corresponding to the melodic phrase of four ascending chords repeating every four seconds. This aspect was further investigated by Eerola et al. (2018) using wavelet and cross-wavelet transform analysis with computer vision tools; the researchers demonstrated a range of periodic behaviours in each performer with frequency peaks that differ for non-pulsed and pulsed jazz duo performances, being at higher frequencies (0.75 and 0.40 Hz, faster movements) for the former, and lower frequencies (0.50 and 0.33 Hz, slower movements) for the latter. The strength of the interperformer interactions in the non-pulsed music, as measured by CWT power, was found to predict audience perception of communicative interactions between co-performers. In a later study, Jakubowski et al. (2020) found that synchrony judgments of jazz duo performances were related to the regularity of the musical pulse, and synchrony ratings increased when musicians’ periodic movements were at similar frequency bands.

CWT has been also used in the context of Indian classical instrumental music, and results show that interpersonal coordination of movements was greater at metrical boundaries and mostly related to cadential than other metrical instances (Clayton et al., 2019). Furthermore, Dotov et al. (2021) found that interpersonal synchrony between audience members, as manifested in their head movements, was tighter with music higher in groove and when audience members could see each other rather than in absence of visual contact, suggesting that social context and musical features impact how the music is embodied. Taken together, these studies demonstrate the successful application of CWT analysis for a better understanding of interpersonal interactions in ensemble playing, by offering new insights into the correspondence between interpersonal coordination and musical features such as structural hierarchy and music styles.

Based on a plurifrequential approach to motion analysis, we investigated interpersonal movement coordination by means of cross-wavelet analysis in piano-singing duos. More specifically, we operationalised the strength of coordination as the power of common periodic oscillations in musicians’ head motion, and the tendency to lead and follow a co-performer in terms of the phase difference between these periodic oscillations. We expected that power and phase difference at nested time scales, from micro structure (i.e., half bar) to macro structure (i.e., four bars and form sections), would reveal the hierarchical nature of head motion in line with the hierarchical structure of the music and the dynamical aspects of leadership.

### The current study

The current study aims to investigate if and how musicians’ body motion coordination relates to the phrase structure of the piece and musicians’ empathy profile. This study focused on Lied duos, i.e. piano-singing duos in which the pianist accompanies the singer performing the melodic line. Lied duos are small ensembles without a conductor, and are of particular interest as they act as self-managed teams, with all ensemble members contributing to the group (D’Amario et al., 2018a; Timmers et al., 2014; Volpe et al., 2016). The duos in the current study rehearsed and performed two songs from the Romantic period. The inclusion of these two pieces allowed us to investigate the evolution of interperformer coordination in a context where the relationships between musicians are dictated by the music to some extent. The following research questions (RQs) and hypotheses were formulated:

1. How does head motion coordination relate to musical structure? We hypothesise that peaks in the similarity between head trajectories correspond to the phrase level of the piece performed, based on previous studies highlighting changes in body motion as a function of the piece structure (Eerola et al., 2018; Jakubowski et al., 2020; Walton et al., 2015).
2. Does musicians’ head motion reflect leader-follower roles induced by the structure of the music performed? We hypothesise that singers’ head motion tends to lead the head motion of pianists. This hypothesis is motivated by research showing that leading and following relationships emerge in the body motion of string quartet musicians when leadership is assigned and manipulated experimentally (Chang et al., 2017). Our hypothesis tests whether similar patterns emerge when leadership is not assigned, but implied by the structure of the music (in Lied duos, singers mostly perform the melodic line and pianists play the accompaniment role; Frăţilă, 2021). We also expect to see that these leader-follower dynamics are stronger in joint sections than in prelude/interlude moments where only the pianist performs.
3. How do the strength and leadership dynamics of musicians’ head motion coordination change with rehearsal? We expect to see that the strength of their similarity becomes greater as more practice is acquired after rehearsing, building on results of previous studies of a string quartet (Wood et al., 2022). Furthermore, in line with results of studies investigating body motion in music duos in the context of rehearsal sessions (Bishop et al., 2019), we also expect that the overall quantity of motion, a measure of the energy of the performance, is greater after rehearsal (when the duo has established familiarity with the music and each other) than before. The musicians’ tendency to lead/follow is hypothesised to be stronger after rehearsal than before rehearsal, as leadership might emerge with time spent interacting and rehearsing.
4. Does EPT impact interperformer coordination? We expect to see that the strength of interperformer coordination, as measured in musicians’ head motion, is higher in more empathic musicians, as it has been shown that empathy promotes note-to-note synchronization skills in individuals without music training (Novembre et al., 2019). We expect to show that higher EPT scores of the singers, who traditionally act as leaders in piano-singing duos, are hypothesised to be related to a stronger tendency to lead.

## Method

### Ethics statement

The experiment was conducted in accordance with the Declaration of Helsinki, and ethical approval for the study, with reference EK Nr: 05/2020, was obtained from the Ethics Committee at mdw—University of Music and Performing Arts Vienna (Austria).

### Participants

A total of 24 participants (age $M=24.9$ years old, SD = 2.3 years old; 14 females, 10 males) took part in the study. Twelve of them were singing students and 12 piano students attending undergraduate and postgraduate courses at the departments of Piano Performance, Chamber Music or Vocal Studies and Music Theatre at mdw. They reported having on average 13.6 years of formal training (SD = 5.6 years) and practicing on average 3.3 h/day (SD =1.2 h). Participants received a compensation of 200 Euros.

### Empathy pre-assessment

Before taking part in the experiment, the empathic perspective taking (EPT) trait of the participants was pre-assessed. EPT was measured using the 7-item “Perspective Taking” sub-scale of the Interpersonal Reactivity Index (IRI) questionnaire (Davis, 1980, 1983), which is the most widely used measure of empathy (Grevenstein, 2020). The EPT sub scale was also provided in a German version (Paulus, 2009) to those participants preferring the German translation to the original English questionnaire. Scores for the German version were then scaled to match the scores of the English version.

The mean EPT score for females was 20.6 (SD = 4.1), and that of males was 19.2 (SD = 3.5). Based on the pianists’ and singers’ median EPT (Mdn = 20, Range = 13–28 for pianists and Mdn = 22, Range = 15–27 for singers), musicians were then split in low (i.e., below the median) and high (i.e., above the median) EPT pianist/singer groups. Then, piano-singing duos were formed by pairing musicians:

- with a similar EPT score within the same EPT group (i.e., absolute EPT difference was not larger than two), and
- with a different EPT score from different EPT groups (i.e., absolute EPT difference $M=6.3$, SD = 2.1),

resulting in the following four groups of 6 duos each:

- LL group (EPT $M=17.1$, SD = 2.3): pianists and singers from the low EPT group
- HH group (EPT $M=23.9$, SD = 1.9): pianists and singers from the high EPT group
- LH group (EPT $M=20.4$, SD = 4.3): pianists from the low EPT group and singers from the high EPT group (absolute EPT difference $M=7.5$, SD = 1.6)
- HL group (EPT $M=20.6$, SD = 3.8): pianists from the high EPT group and singers from the low EPT group (absolute EPT difference $M=6.1$, SD = 2.3)

### Materials

Two contrasting pieces for voice and piano from the Western classical musical tradition were used in the study: Automne Op. 18 N. 3 composed by Gabriel Fauré (presented in the original key of b minor and in the transposed key of c sharp minor) and Die Kartenlegerin Op. 31 N. 2 composed by Robert Schumann (presented in the original key of E flat major and in the transposed key of F major). Full scores of the Fauré and Schumann piece in the original key are reported in Supplementary material Figs. 1 and 2, respectively.

Both pieces are from masters of the Lied duo repertoire of the XIX century, featuring a distinct role distribution between musicians, with the pianist being the accompaniment of the singer performing the melodic line (Frăţilă, 2021). Nevertheless, the pieces differ substantially in melodic contour and harmonic structure, with the Fauré piece being slower and with a more stable tempo. The pieces also differ in their formal structure, being a clear *A*, *B* and *A*′ structure for the Fauré piece and more elaborated (*A*, *A*′, *B*, *A*″, *C*, *A*′′′) for the Schumann piece (form analysis reported in Table 1a and b, respectively). Whilst the piano in the Fauré piece always accompanies the singer, the piano in the Schumann piece sometimes plays the melody alongside the singer (e.g., bar 17, Supplementary material Fig. 2), thus contributing to the performance of the leading theme. The Schumann piece features several solo sections (as shown in Table 1a) and tempo changes (e.g., *rit.* at bars 17–19, 25, 36–38, etc.), which were challenging for duos to coordinate. The Fauré piece also presents solo moments, but fewer in number, as shown in Table 1b. The Fauré piece comprises 39 bars and the performance was on average 169 s long; the Schumann piece consists of 156 bars and its performance was on average 201 s long. The selection of pieces for the study was deliberated with the aid of a professional pianist and a professional singer. They were chosen because they pose diverse challenges for the performers’ coordination, while still being feasible to master during a short rehearsal session. Singers were allowed to choose the key for each piece that better fits their singing range.

**Table 1:**
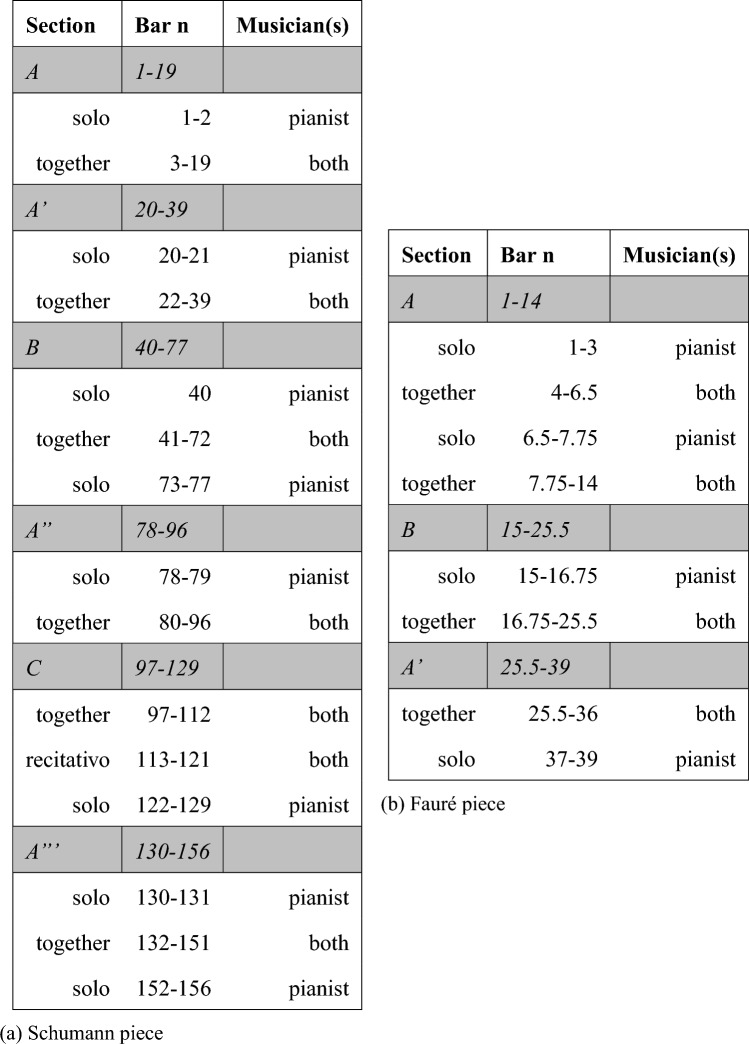
Form analysis of the pieces used in the study: (a) the Schumann piece and (b) the Fauré piece

Labels and bar *n* of the main sections are presented in italics

### Design

This study used a 2 (rehearsal sessions) $\times$ 2 (pieces) $\times$ 4 (takes, take 0 pre-rehearsal and takes 1, 2 and 3 post-rehearsal) $\times$ 4 (EPT group) $\times$ 6 (duos) design, comprising a total of 24 duos and 8 repeated performances recorded per duo. Take 0, being the first time duos performing together, and take 3, the last performance after rehearsals, were expected to show most contrasts in interperformer coordination patterns; take 2 was also analysed to investigate the consistency of any results found in take 3. Take 1 was expected to be more similar to takes 2 and 3 than take 0, so to reduce the number of statistical tests, we excluded Take 1 from our analyses.

The order of rehearsing and performing the two pieces was counterbalanced across sessions and EPT groups. Measures of interperformer coordination (i.e., power and phase difference of the CWT) were the response variables, whilst empathy measures (i.e., EPT group and musicians’ own EPT score) were the explanatory variables. Duo number was the random variable.

### Apparatus

The experiment took place in a seminar room of the Department of Music Acoustics at mdw—University of Music and Performing Arts Vienna (Austria). Each pianist performed sitting at the piano bench and each singer stood in front of the piano facing the experimenters (and imagined audience), as if they were performing on stage.

Three sets of data were collected: (1) audio from the piano, from a head-mounted close proximity microphone (Sennnheiser ew 100 G2) and two stereo condenser microphones (Neumann KM A P48) providing right and left outputs; (2) MIDI from a Yamaha Clavinova; and, (3) body motion data from an optical motion capture system.

The head-mounted microphone was placed on the cheek of the singer approximately 2.5 cm from the lips. The stereo microphones pointed towards the pianist and the singer. Four audio outputs (1 head-mounted microphone, 1 mono audio from the piano, 2 stereo microphone picking room sound) fed into a multi-channel audio interface (Focusrite Scarlett 18i8), which was connected to a PC. The 4 outputs, along with MIDI from the piano, were then recorded using a digital audio workstation (Ableton Live) as separate tracks at a sampling frequency of 44.1 kHz and 32-bit depth.

Body motion was recorded at 240 Hz using a 12-camera (Prime 13) OptiTrack motion capture system. Pianists’ body motion data consisted of trajectories from 14 reflective markers placed on the head and upper body, as follows: 3 markers on the head, 2 on the back, 1 per shoulder, arm, wrist, and hand, and 1 on the chest. Singers’ body motion data included trajectories of four extra markers placed around the hip, for a total of 18 markers.

Audio recordings were synchronized with OptiTrack recordings using an audiovisual signal produced by a film clapboard, marked with reflective markers and placed in view of the motion capture cameras, near to the two stereo microphones collecting audio from the room. The clapboard was struck at beginning of each recording, and all recordings were synchronized retrospectively to this point. Video recordings of rehearsal sessions, eye-gaze/pupillometry data from participants, and breathing and cardiac activity during the ensemble performances were also collected but are not part of the current report.

### Procedure

Participants were invited to take part in two sessions within the same week. Each session was about 2 h long. At the beginning of the first session, participants received spoken and written explanations of the research project and the tasks, then they gave written consent to take part in the study. In each session, they rehearsed each piece for about 10 min with their assigned duo partner, and performed them one time before and three times after rehearsal. Post-rehearsal performances were blocked by piece; that is, the duos played three times through one piece, then three times through the other piece. They had about 1 min rest between performances within blocks and about 2 min rest between blocks.

Musicians were instructed to use the rehearsal time to prepare a camera-ready interpretation of each piece, and to give their post-rehearsal performances as though on a concert stage. These instructions were meant to encourage naturalistic behaviour from the musicians and high-quality, musically expressive performances.

Musicians received a digital copy of the scores one week prior to the experiment to practice and learn the pieces by themselves, but were not allowed to practice with their co-performers outside of the experimental sessions. Co-performers within the same duo did not know each other before the experimental session took place, and met for the first time in the laboratory. This was done so that we could record changes in the development of interperformer interactions that emerged as a result of their exchanges in the laboratory. Musicians played from scores placed on the music stand; some of them used the paper copies, others the digital versions displayed on their own personal tablet. They were not aware of the purpose of the study and an audience was never present.

### Analysis

Interperformer coordination in piano-singing duo performances was measured using cross wavelet transform (CWT) analysis and investigated in relation to the musical structure and the empathic profile of the musicians, by implementing a three-step analytical approach as follows:

1. Musical tempo computation:
  - Alignment of MIDI and score.
  - Computation of average inter-beat-intervals (IBI) from pianists’ MIDI data.
  - Calculation of mean duration of multiple hierarchical phrase levels (i.e., half bar, one bar, two bars, three bars, and four bars) for each take/piece/duo.
2. Power and phase difference of head motion coordination across a broad frequency range:
  - Extraction of the front head marker position from the MoCap data.
  - Computation of 3d velocity trajectories of the front head markers of each musician.
  - Extraction of the CWT power and phase difference on the chosen broad-band.
3. Analysis of musicians’ coordination strength and leading/lagging behaviours across phrase levels:
  - Computations of CWT power and phase difference in selected narrow-bands corresponding to the different hierarchical phrase levels.
  - Analysis of CWT power and phase difference as a function of phrase levels, and identification of the most dominant phrase level, measured as grand average of CWT power.
  - Analysis of the impact of piece, take and musical section on CWT power and phase difference at the most dominant phrase level.
  - Analysis of the impact of the empathic profile of the musicians on mean CWT power and phase difference, in relation to the whole take, to different music sections, and to solo versus together moments.

**Fig. 1:**
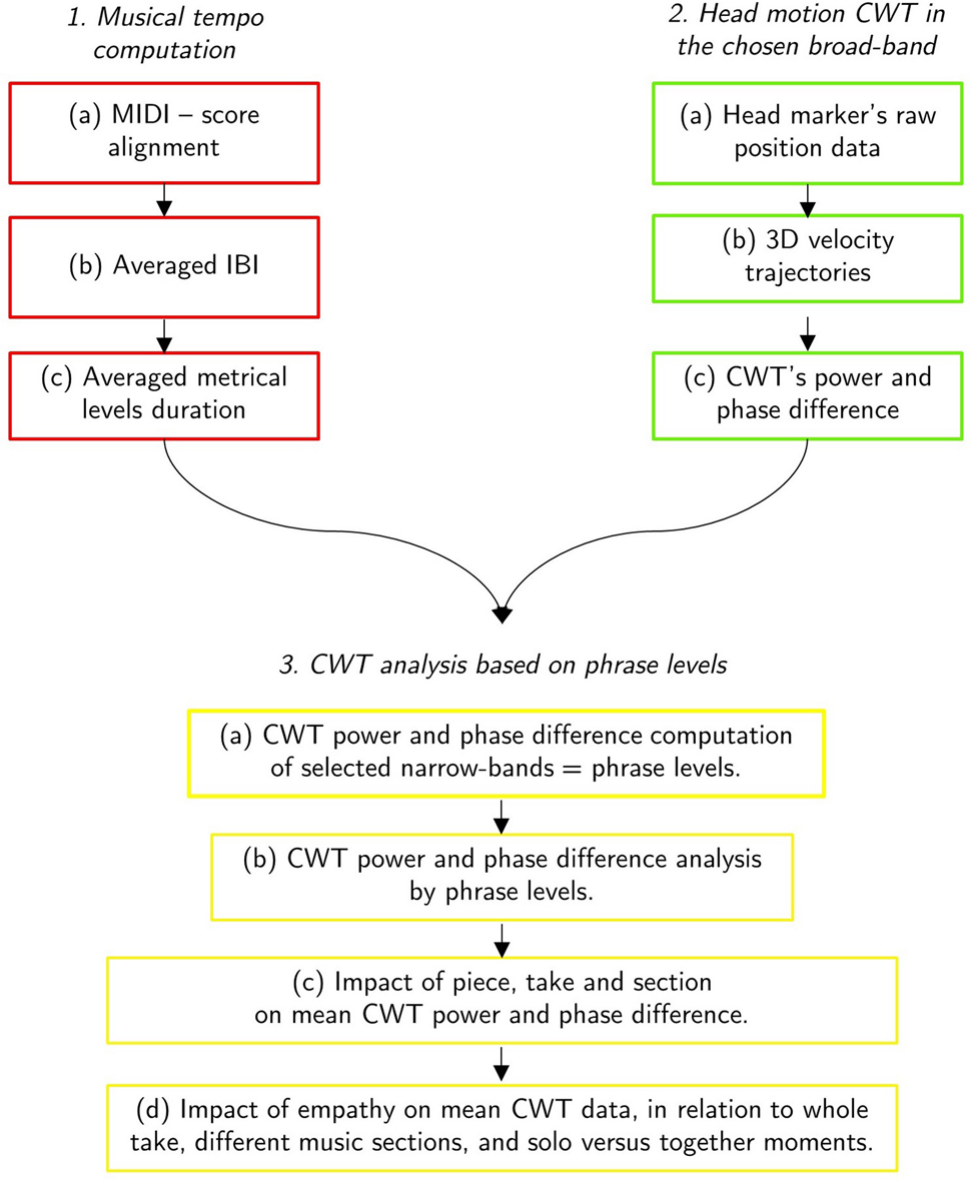
Flow chart of the stepwise analytical framework used for the analysis of interperformer coordination by means of cross wavelet transform (CWT) power and phase difference in motion of the front head markers placed on both musicians. In addition to these steps, the analysis also included computation of the overall musicians’ quantity of motion, in order to measure the overall energy of the performance as related to a total of 14 markers applied to each musician

A flowchart of this approach is shown in Fig. 1 and the details are provided step-by-step in the sections that follow.

#### Step 1: Musical tempo computation

MIDI data from the Clavinova were aligned to digital versions of the scores using the performance-score matching algorithm developed by Nakamura et al. (2017) (Fig. 1, step 1a). Output was parsed and alignments were corrected using the Python package “partitura” (Grachten et al., 2019) and Parangonada interface.^2^ Alignments were then imported into R, and tempo curves were obtained for each performance by computing the average onset time per beat and the series of intervals (in seconds) between each beat onset. Wherever a beat had no performed onsets (either because the score contained a rest or because the performer missed a note), a linear interpolation was run to estimate the missing beat times. An averaged inter-beat interval (IBI) was then computed per performance as the measure of musical tempo (step 1b).

**Fig. 2:**
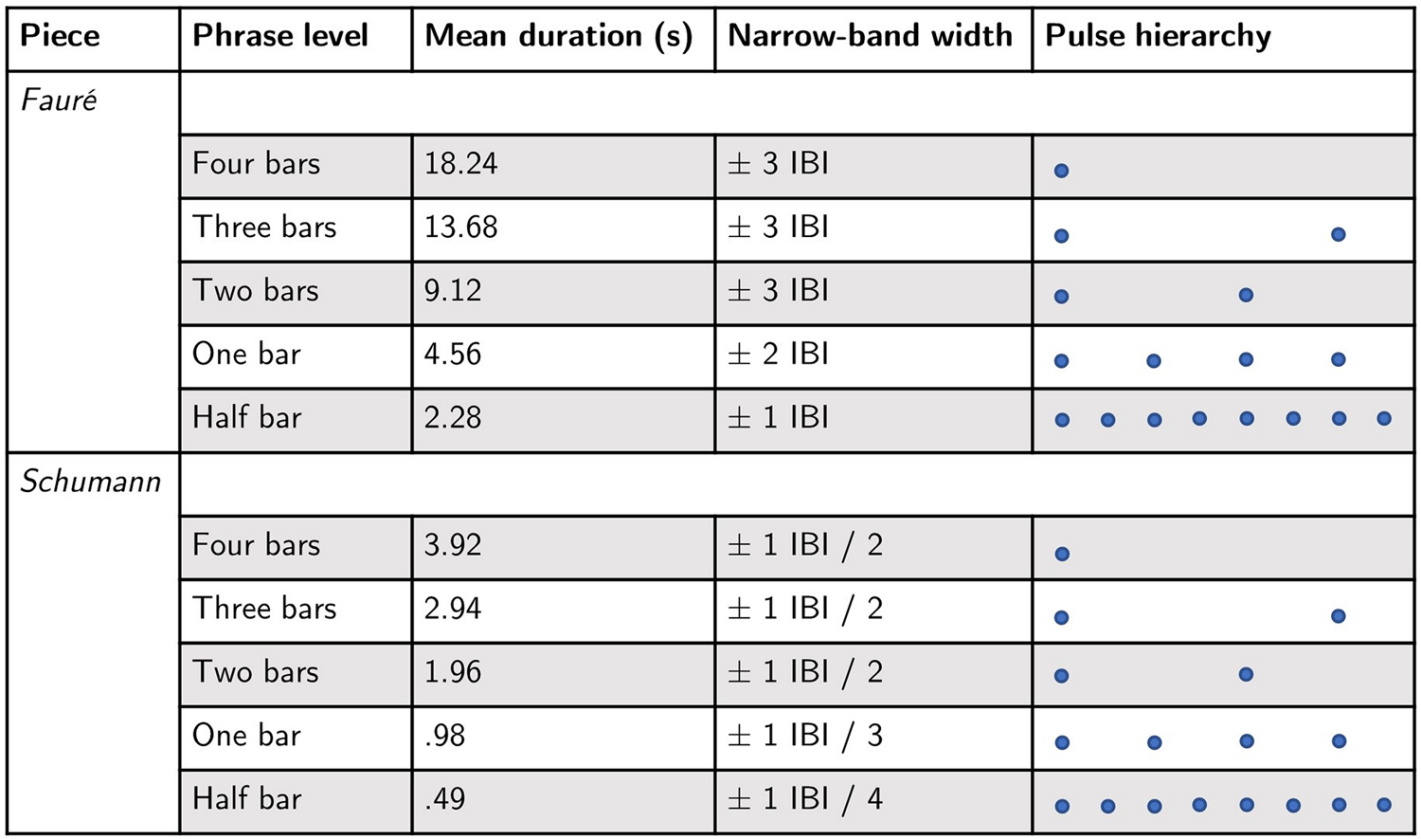
Display of the phrase levels of the Fauré and Schumann piece, in relation to the corresponding mean duration, width of the CWT narrow-band, and pulse hierarchy. The narrow-band width was computed based on the inter-beat-interval (IBI) calculated for each individual take/piece/duo

The meter is 12/8 in the Fauré piece (compound quadruple meter) and 2/8 in the Schumann piece (simple duple meter). Therefore, for the purpose of analysis, beats were measured at the 1/8 note level for both pieces (acknowledging that performers may not have felt the beat at this fine-grained level; see below). The mean IBI for Schumann across all performances was 0.49 s (SD = 0.04 s, range = 0.4–0.6 s) and the mean IBI for Fauré was 0.38 s (SD = 0.03 s, range = 0.32–0.5 s). Finally, mean duration of different hierarchical phrase levels (half bar, one bar, two bars, three bars, and four bars) were computed for each piece and performance based on the mean IBI of that performance (step 1c). These values are reported in Fig. 2.

#### Step 2: Power and phase difference of head motion coordination across a broad frequency range

The analysis of interperformer coordination focused on head motion, which is closely tied to visual expressivity (Bishop et al., 2019; Glowinski et al., 2013; Goebl & Palmer, 2009; Keller & Appel, 2010).

Front head marker position data were smoothed and velocity was derived using a Savitzky–Golay filter (polynomial order 3, window size 25), through the “prospectr” package (Stevens & Ramirez-Lopez, 2021) in R (R Core Team, 2013)—(step 2a). Then, the Euclidean norm of 3D data was calculated (step 2b). The analysis was based on velocity, which can be conceptualized in terms of “quantity of motion” (i.e., displacement per unit of time) or motion energy or intensity. Velocity is commonly used as a measure of motion in studies of musical movement (Bishop et al., 2021; Ragert et al., 2013; Timmers et al., 2014) because, unlike position, its values do not depend on the specific orientation of the performer in the motion capture volume, and it is less affected by noise than are other derivatives of position (e.g., acceleration and jerk; see van Dorp Skogstad (2014) pp 30–31 for a mathematical explanation).

Next, velocity trajectories were subject to cross-wavelet transformation (CWT) for each duo and take (i.e., take 0, before rehearsal, and takes 2 and 3, after rehearsal), using the R package “WaveletComp” (Roesch & Schmidbauer, 2018) with the complex-valued Morlet wavelet as mother wavelet. The range of periods to be considered was set in line with the phrase structure of the pieces, and ranged from about one half bar to 4 bars. The upper value of this range was calculated using the slowest mean IBI identified across duos and takes, whilst the lower value of the period range was calculated using the fastest mean IBI identified across duos and takes. Thus, the range for the Fauré piece was from 1 to 26 s, and the range for the Schumann piece was from 0.2 to 6.2 s. From now on, this broad range of frequencies will be referred to as “broad-band”.

**Fig. 3:**
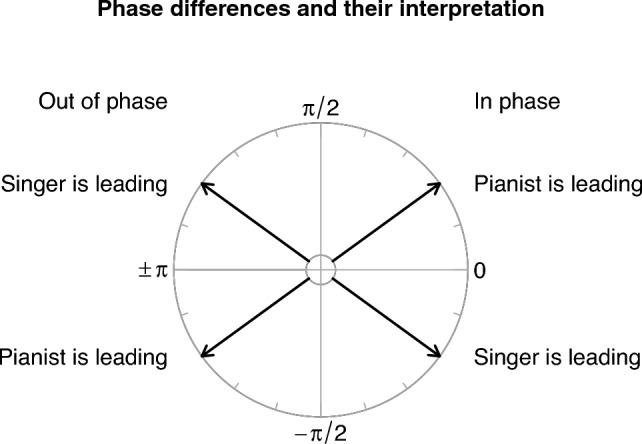
Scheme to interpret phase differences between the pianist and singer in a duo, referring to the period in question. The period is represented by the full circle. Phase difference is measured as angles in the interval from $-\pi$ to $\pi$. Zero phase difference indicates that the two corresponding velocity series are perfectly in sync; while the series move in perfect anti-phase for values of $\pm \pi$. The relation of the two series is still called in-phase or out-of-phase when shifting away from these two extremes by less than a quadrant, i.e. less than a quarter of the period. Phase difference values in the intervals $[0, \pi /2]$ and $[-\pi ,-\pi /2]$ indicate that the pianist is leading and the singer is following; conversely, values in the intervals $[\pi /2, \pi ]$ and $[-\pi /2, 0]$ show that the singer is leading and the pianist is following. The R package “WaveletComp” (Roesch & Schmidbauer, 2018) used for the analysis received the pianist’s time series as the first input, and the singer’s as second input; the roles of “leader” and “follower” swap with the input order.

From the cross-wavelet transformation of 3D velocities across broad-band frequencies, the power spectrum was extracted as a measure of the strength of coordination between musicians in head motion in the time-period domain (step 2c).^3^ Phase differences between musicians’ head velocity oscillations were also extracted as a measure of leading and lagging. This information is given in terms of angles in a cycle, as shown schematically in Fig. 3.

#### Step 3: Analysis of musicians’ coordination strength and leading/lagging behaviour across phrase levels

Using the power and phase information computed for broad-band frequencies, five non-overlapping “narrow-bands” were then defined. These narrow bands included a range of frequencies with periods corresponding to approximately one half bar, one bar, two bars, three bars, and four bars (step 3a). Narrow bands were computed individually for each duo, piece, and take, using the corresponding average IBI. A scaled width for each narrow-band was defined for the two pieces, as shown in Fig. 2.

Then, for each period within the narrow-bands, time-series data of power and phase difference per observation across the duration of the performance were extracted, and grand averages were computed across timestamps and period range for each narrow-band, duo, and take. Two linear mixed models were implemented to compare CWT power between narrow-bands (phrase levels) and identify the most dominant phrase level(s) for each piece and take across the 24 duos, i.e. the phrase level with the highest CWT power (step 3b).

Eventually, several different models were set up to study the impact of piece, take, piece section, and the musicians’ empathic profile on CWT power and phase difference, as measures of interperformer coordination (steps 3c–d).

### Robustness checks

Model residuals were found to pass the test for homoskedasticity in all cases, using the R package “DHARMa” (Hartig, 2021). However, the results of CWT analysis presented above are based on a series of decisions (e.g., choice of the mean rather than the median), which might have given rise to several issues; these issues are addressed in the Supplementary material (see Supplementary Material Analysis) with the aim of validating the robustness of the results. Robustness checks covered alternative velocity data processing and outliers sensitivity of results. In summary, all checks supported our findings.

## Results

This section reports the results of cross-wavelet transform (CWT) analysis by phrase levels (step 3), aiming at: (1) analysing CWT power and phase difference by phrase level (steps 3a-b), (2) analysing CWT output by piece/take/section (step 3c), and (3) investigating the impact of the musicians’ empathic perspective taking (EPT) on CWT power and phase difference (step 3d). The section concludes with the analysis of the overall quantity of motion of the performance, providing an additional feature of interperformer coordination, and a report on the outcome of various robustness checks, aiming at validating the results.

### Coordination strength and leading/lagging by phrase level

**Fig. 4:**
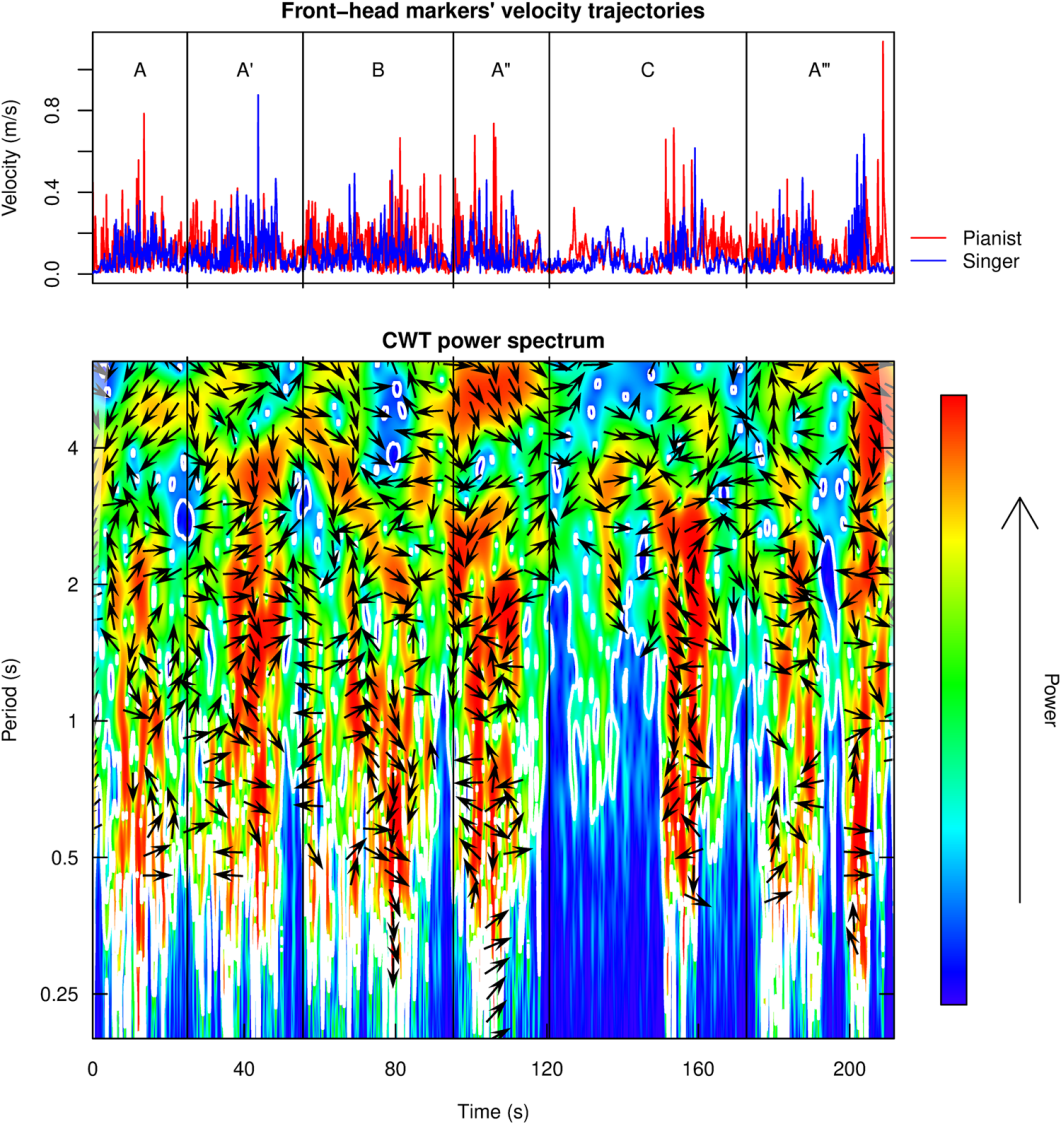
Example heat plot of the CWT power spectrum (bottom) as computed from the front-head marker velocities (top) for a duo performing the Schumann piece for the third time after a rehearsal session. Arrows indicate the CWT phase difference, as described in Fig. 3. Vertical lines identify piece section boundaries, as listed in Table 1. The segmentation of the CWT data in line with the score was mostly based on the MIDI data, providing note-by-note time stamps. Unfortunately, eight out 72 MIDI recordings were corrupted; the segmentation in this case was manually computed (by the first author) by extracting the relevant time-stamps from the room microphones using Audacity (Audacity, 2021)

Step 3a: In order to investigate interperformer coordination, the CWT power spectrum of the front-head markers’ velocity trajectories was computed for each duo, take and piece, within the chosen broad-band of 1–26 s for the Fauré piece and 0.2–6.2 s for the Schumann piece. Figure 4 provides an example image plot of the power spectrum in the time-period domain, as extracted for one performance of the Schumann piece, together with the performers’ velocity trajectories. While the horizontal axis monitors the time elapsed (in s), the vertical axis (on a logarithmic scale) refers to the chosen broad-band of potential periodic components in the two trajectories. The color bar reveals the gradient of power with which a period occurs jointly, white contour lines delineate areas of significance. The plot also features information about the performers’ phase difference in the time-period domain, and thus their tendency to lead/lag, depicted by arrows according to the scheme in Fig. 3

**Fig. 5:**
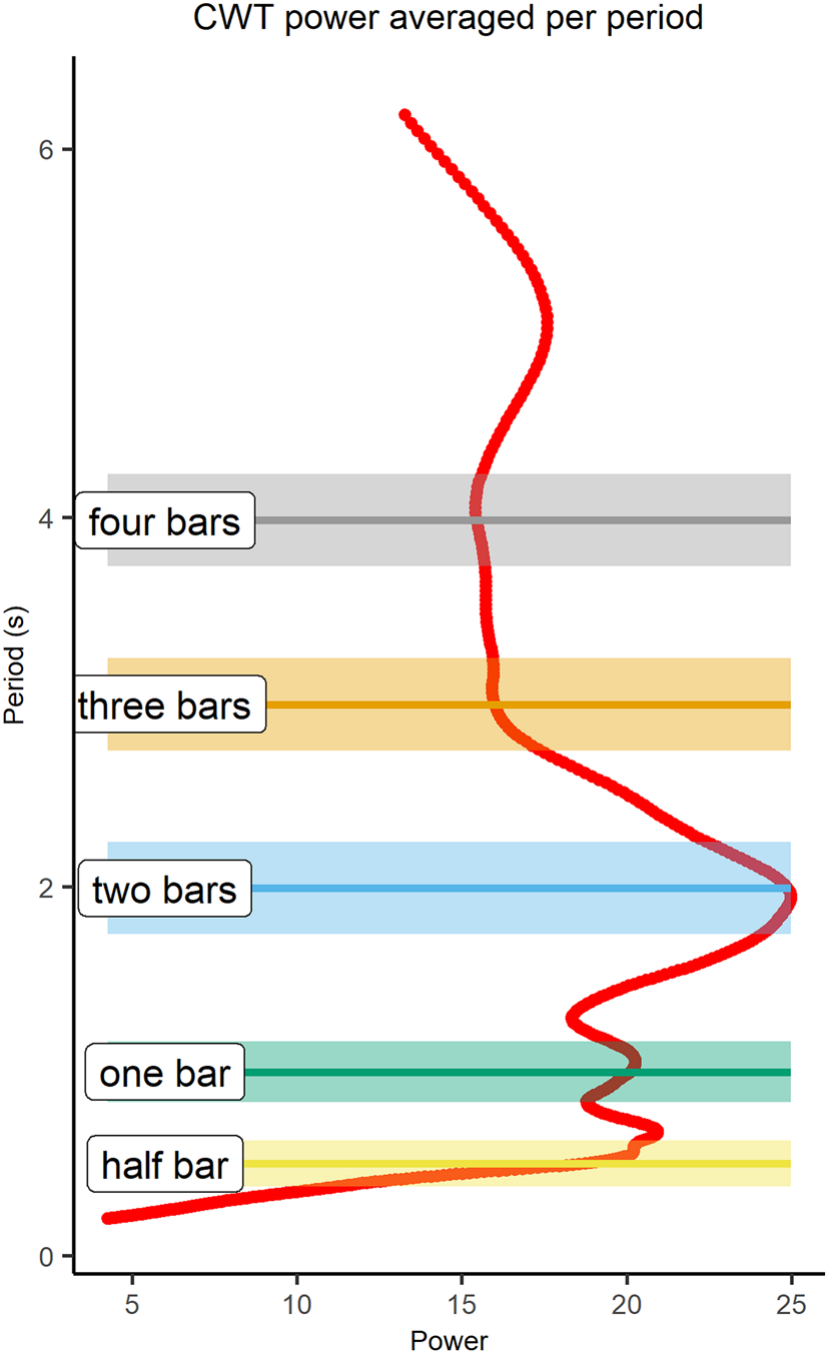
Example of a CWT power (red line) averaged across time per period in the Schumann piece, take 3, plotted against the non-overlapping phrase levels of a given duo (i.e. half bar, one bar, two bars, three bars and four bars duration), estimated based on the individual inter-beat interval (IBI). Visual inspection of this graph reveals a tendency for CWT power peaks to correspond to the phrase levels, and for mean CWT power to lower with phrase levels longer than two bars

For each period within the chosen broad-band, CWT power was averaged across time and analysed in relation to the individual phrase levels (i.e., half bar, one bar, two bars, three bars and four bars, computed based on the respective duo’s own IBI), as shown in the example in Fig. 5. An analysis of the number of occurrences of the average power peaks (at least out of the three highest) that fell inside the duration range of one of the individual phrase levels was done for each duo/take/piece. As shown in Table 2, most of the peaks fell into the half-bar and one-bar range in case of the Fauré piece, while two bars were found most prominent in case of the Schumann piece. Few peaks were also found within the remaining phrase levels. These findings suggest that interperformer coordination occurs predominantly within the musical phrase levels.

**Table 2:** Occurrence of one of the first three CWT power peaks by phrase level. The occurrence is expressed as percentage of the total number of peaks falling in one of the phrase levels

Piece	Half bar	One bar	Two bars	Three bars	Four bars
Fauré	46	43.5	3.5	3.5	3.5
Schumann	14	23	44	17	4

Step 3b: Next, two linear mixed-effects models (i.e., one for each piece) were fitted (estimated using Restricted Maximum Likelihood, REML, and nloptwrap optimizer) to measure the impact of phrase levels on power data. This also identified the phrase level with the strongest CWT power (i.e., the most dominant phrase level). The strength of the CWT power might be related to the tempo of the overall performance, and for this reason we decided to run a model for piece, as they differed in average tempo. CWT power as response variable was aggregated by phrase level and take per duo. Take number was nested under phrase level and duo number was entered as random effect. The power model formula used for each piece was:

$$\begin{aligned} \mathrm{{power}}\sim \mathrm{{phrase}}\_\mathrm{{level/take}}. \end{aligned}$$

**Fig. 6:**
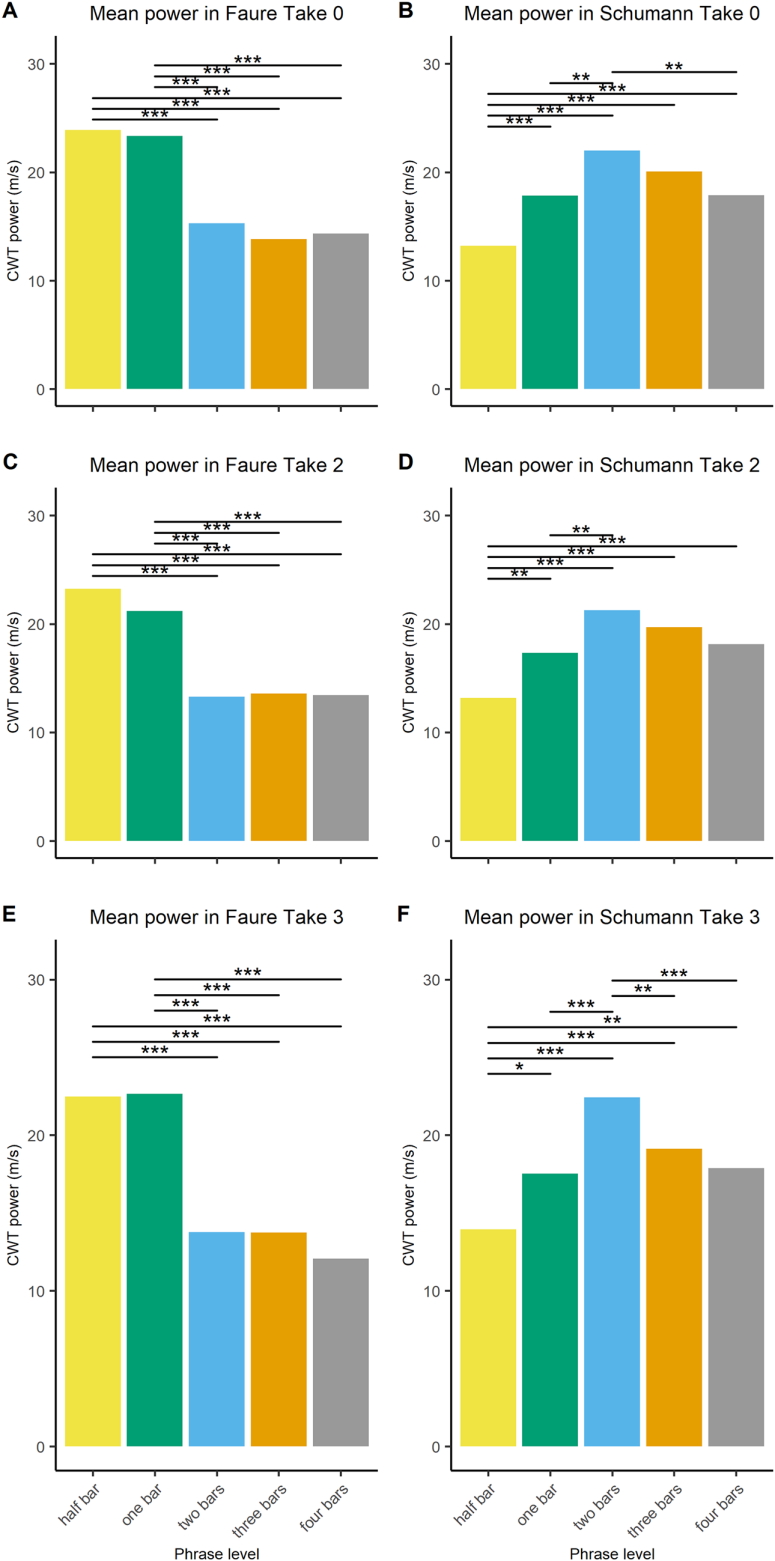
Grand mean power by different phrase levels (i.e., half bar, one bar, two bars, three bars, and four bars) for the three takes (i.e., take 0, take 2 and take 3) of the Fauré piece (**A**, **C**, **E**) and the Schumann piece (**B**, **D**, **F**). Horizontal lines display the significant outcomes from the Tukey test of equal means; significance codes: $$^{***}$$ 0.1%, $$^{**}$$ 1%, $$^*$$ 5%

For the Fauré piece, the model’s total explanatory power was substantial (conditional $R^2 = 0.55$) and the part related to the fixed effects alone (marginal $R^2$) was 0.43. Results demonstrate that, regardless of the take number, CWT power was stronger at the half bar level $(\beta = 9.5,\; 95\%\; \mathrm{{CI}} \,[6.9, 12.2],\; t(343) = 7.1,\; p < 0.001)$ and the one bar level $(\beta = 9,\; 95\%\; \mathrm{{CI}} \,[6.3, 11.6],\; t(343) = 6.6,\; p < 0.001)$ compared to the four bar level, set as baseline. Interestingly, post-hoc comparisons using the Tukey test indicated that the half bar level was most prominent for take 0 and 2, whilst for take 3 it was the 1 bar level (see Fig. 6A, C, E).

For the Schumann piece, also, the model’s total explanatory power was substantial (conditional $R^2 = 0.49$) and the part related to the fixed effects alone (marginal $R^2$) was 0.37. Results demonstrate that, regardless of the take number, CWT power was significantly stronger at the two bar level $(\beta = 4.1,\; 95\%\; \mathrm{{CI}} \,[2.2, 6],\; t(343) = 4.3,\; p < 0.001)$ and the three bar level $(\beta = 2.2,\; 95\%\; \mathrm{{CI}} \,[0.3, 4.1]$, $t(343) = 2.3,\; p < 0.05)$ compared to the four bar level, set as baseline (see Fig. 6B, D, F). Post-hoc Tukey comparisons demonstrate that for take 3, the two bar level was more prominent even than the three bar level (see Fig. 6F).

Figure 6 provides a summary of the impact of phrase level on CWT power for the Fauré piece (Fig. 6A, C, E) and the Schumann piece (Fig. 6B, D, F). Table 3 presents the period range pertaining to the greatest grand mean power for each take/piece, which from now on will be referred to as the most dominant phrase level. Taken together, these results demonstrate that the phrase level had an impact on the CWT power, which was strongest at half bar (takes 0 and 2) and one bar (take 3) in the Fauré piece, and at about two bars in the Schumann piece (all takes).

**Table 3:** Summary of the most dominant phrase level computed for each take and piece, featuring related phrase level, and mean CWT power, mean (SD) signed CWT phase difference (transformed from radians into ms), leadership, and phase direction

Piece	Take	Most dominant phrase level	Mean CWT power	Mean (SD) CWT phase difference (ms)	Leadership	Phase direction
Fauré	0	Half bar	23.88	396 (539)	Pianist	In phase
	2	Half bar	23.22	$-$ 128.3 (873)	Singer	In phase
	3	One bar	22.65	$-$ 1050 (2175)	Singer	In phase
Schumann	0	Two bars	22	$-$ 108 (753)	Singer	In phase
	2	Two bars	21.3	87 (719)	Pianist	In phase
	3	Two bars	22.4	$-$ 136 (683)	Singer	In phase

To test the effect of phrase level on phase differences (musicians’ leading/following behaviour), two other linear mixed models (i.e., one per piece) were fitted. CWT phase difference data aggregated by phrase level and take per duo were entered as response variables, with take number nested under phrase level as fixed effect. The phase difference model formula was as follows:

$$\begin{aligned} \mathrm{{phase}}\_ {\rm{difference}} \sim \mathrm{{phrase}}\_\mathrm{{level/take}}. \end{aligned}$$

**Fig. 7:**
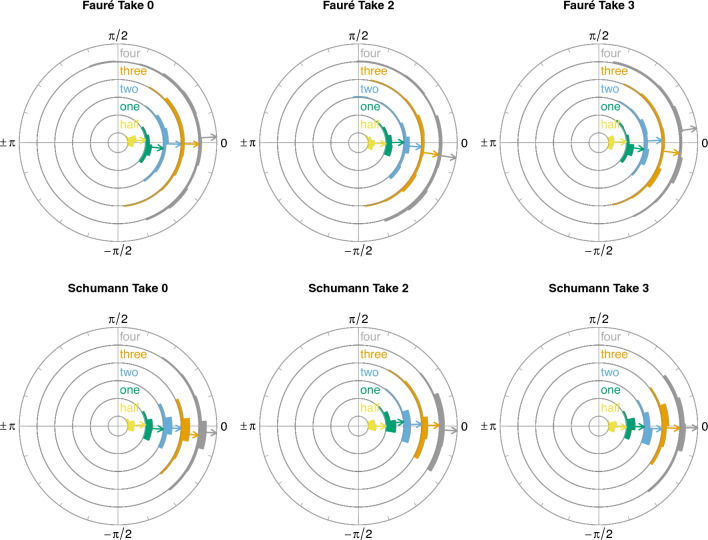
Grand mean CWT phase differences between pairs of musicians’ head velocity trajectories

For the Fauré piece, the model’s total explanatory power was weak (conditional $R^2 = 0.13$) and the part related to the fixed effects alone (marginal $R^2$) was 0.02. For the Schumann piece, the model’s total explanatory power was weak (conditional $R^2 = 0.02$) and the part related to the fixed effects alone (marginal $R^2$) was 0.02. Results show no significant effect of phrase level on CWT phase difference, neither for the Schumann piece nor the Fauré piece. Musicians’ head motion was, on average, in-phase in all takes and both pieces, as shown in Fig. 7 (produced using R package “circlize”; Gu et al., 2014).

### Impact of piece, take and section

Step 3c: Two models tested the impact of piece and take on mean CWT power and phase difference, respectively. For CWT power, a linear mixed-effects model was fitted (estimated using Restricted Maximum Likelihood, REML, and nloptwrap optimizer), using the R package “lme4” (Bates et al., 2015). The power model formula was as follows:

$$\begin{aligned} \mathrm{{power}}\sim \mathrm{{piece/take}}. \end{aligned}$$

**Fig. 8:**
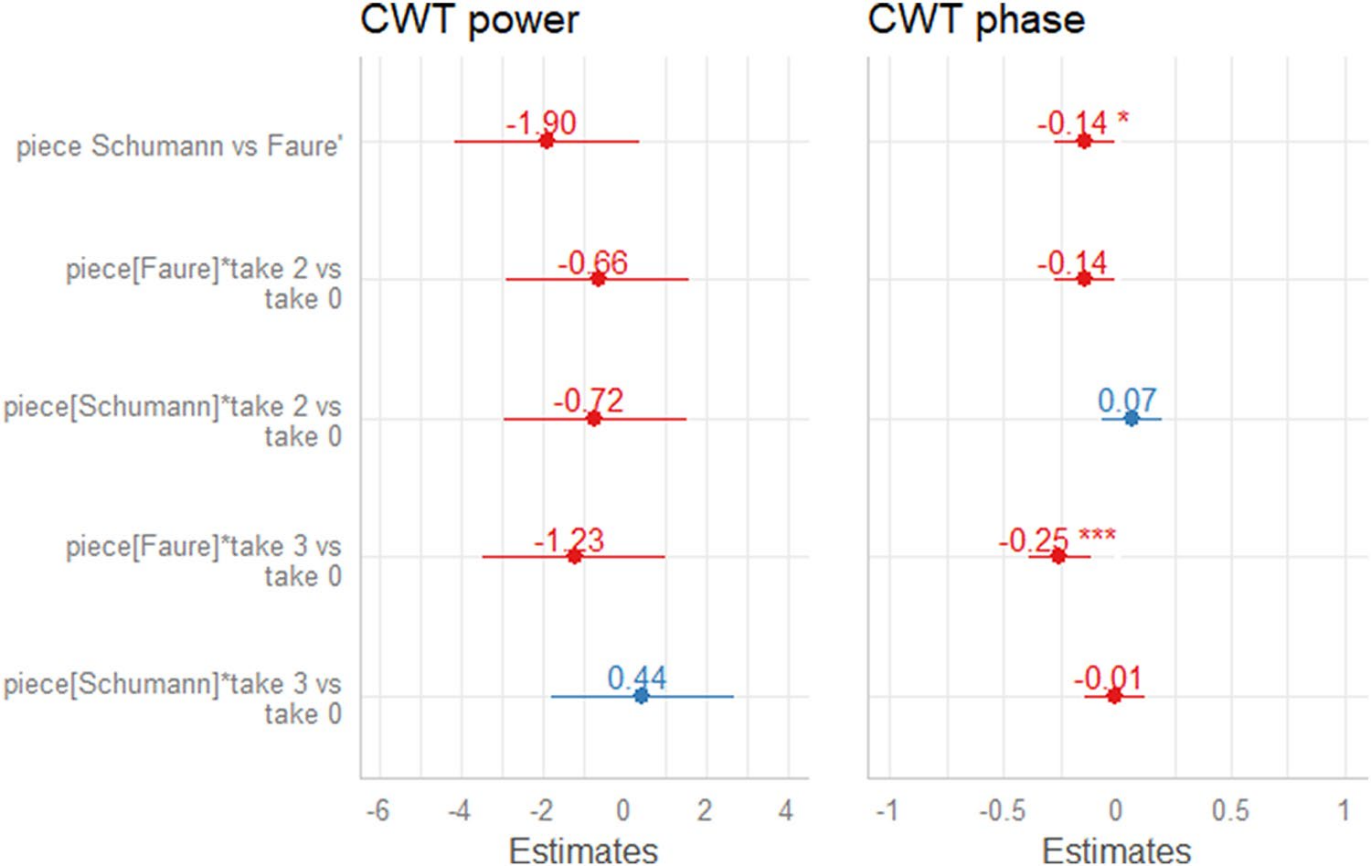
Estimates of piece and take effects (the latter nested under piece) related to mean CWT power (left side plot) and phase difference (right side plot). Piece estimates are given above with reference to the specified base level of the factor (i.e., Schumann versus Fauré, take 2 versus take 0 and take 3 versus take 0). The Tukey method has been used for adjusted *p* values. Significance codes: $$^{***}$$ 0.1%, $$^{**}$$ 1%, $$^*$$ 5%

The model included CWT power at the dominant period range identified in Step 3b, aggregated per duo by piece and take, as the response variable. This allowed us to enter piece as a fixed effect. To measure the impact of repeated performances of the same piece, take was also entered nested under piece. Finally, duo number was entered in the model as random effect. The model’s total explanatory power was substantial (conditional $R^2 = 0.32$) and the part related to the fixed effects alone (marginal $R^2$) was 0.03. Neither piece nor take was a significant predictor of CWT power (Fig. 8). In other words, no significant difference in the strength of the interperformer coordination was detected across pieces nor across repeated performances within the same piece.

For phase difference, a linear model was fitted (estimated using Ordinary Least Squares, OLS), to predict the effect of take, nested within piece, on CWT phase difference at the dominant phrase level (i.e., CWT power), aggregated per duo by piece and take. The phase difference model formula was as follows:

$$\begin{aligned} \mathrm{{phase}}\_ \mathrm{{difference}} \sim \mathrm{{piece/take}}. \end{aligned}$$

In this model, duo number was initially entered as random effect, but dropped because the variance was negligible ($<0.001$), and including it in the model generated a singular error. Thus, a linear model was chosen and run using the lm function from the “stats” package, which is basic R functionality. The model explained a moderate proportion of variance $(R^2 = 0.10,\; F(5, 138) = 3.13,\; p =.010,\; {\rm{adj.}}\; R^2 = 0.07)$. The tendency for the singer to lead was weaker in the Schumann piece $(\beta = -0.14,\; M = -0.1,\; 95\%\; \mathrm{{CI}} \,[-0.28, -0.03],\; t(138) = -2.12,\; p < 0.5)$, than in the Fauré piece $(M = -0.3,\; 95\%\; \mathrm{{CI}} \,[-1.14, -0.04])$. Take number predicted CWT phase difference depending on the piece being performed: it was not a significant predictor in the Schumann piece, but it was a significant predictor in the Fauré piece. As shown in Fig. 8, the tendency for the singer to lead and pianist to follow was higher in the last take $(\beta = -0.25,\; M= -0.15,\; 95\%\; \mathrm{{CI}} \,[-0.39, -0.12],\; t(138) = -3.74,\; {p_\mathrm{{adj}}} < 0.001$), than in the first take $(M = 0.1,\; 95\%\; \mathrm{{CI}} \,[-1.59, -0.49])$. Thus, the leader-follower relationships between co-performers differed depending on the piece being performed and across repetitions. Singers tended to lead and pianists tended to follow more pronouncedly in the Fauré piece than the Schumann piece, and more pronouncedly in take 3 than take 0 of the Fauré piece.

In addition to the last two analyses conducted on the take as a whole, CWT power and phase difference were also analysed as a function of the solo versus together sections for each piece/take at the dominant phrase level, using a model for each CWT measure. The power and phase difference model formulae were, respectively, as follows:

$$\begin{aligned}{} & {} \mathrm{{power}} \sim \mathrm{{solo}}\_\mathrm{{together}} \\{} & {} \mathrm{{phase}}\_\mathrm{{difference}} \sim \mathrm{{solo}}\_\mathrm{{together}} \end{aligned}$$

Solo versus together sections predicted CWT power in all takes of the Fauré piece, and CWT power was higher in the together sections (take 0: $\beta = 6,\; M= 26,\; 95\%\; \mathrm{{CI}} \,[1.9, 11.8],\; t(140) = 2.8,\; p < 0.01$; take 2: $\beta = 11.1,\; M= 27,\; 95\%\; \mathrm{{CI}} \,[7.1, 15.2],\; t(140) = 5.4,\; p < 0.001$; take 3: $\beta = 9.4,\; M= 25.76,\; 95\%\; \mathrm{{CI}} \,[5.2, 13.6],\; t(140) = 4.4,\; p < 0.001$) than the solo parts (take 0: $M = 19.8,\; 95\%\; \mathrm{{CI}} \,[0.1, 0.8]$; take 2: $M = 15.9,\; 95\%\; \mathrm{{CI}} \,[0.5, 1.1]$; take 3: $M = 16.2,\; 95\%\; \mathrm{{CI}} \,[0.4, 1]$). However, solo versus together parts did not predict CWT power in any of the Schumann takes. Interestingly, results demonstrate that phase difference did not change across solo/together sections, regardless of the piece and take, demonstrating that the leader–follower relationships between musicians were unchanged during the piano solo sections.

In summary, these results indicate that the tendency to lead/lag a co-performer was impacted by the piece being performed and the repeated performances. The tendency for the singer to lead and the pianist to follow was stronger in the Fauré piece (more rhythmically stable), and leadership patterns were stronger at the ending of the laboratory session than before rehearsing in the case of the Fauré piece. The strength of interperformer coordination, as measured by the CWT power, did not change across piece or take, but was stronger in the together sections than in the piano solos of the three takes of the Fauré piece.

### Impact of empathy on interperformer coordination

#### Whole piece

Step 3d: Having shown that coordination strength (as measured by CWT power) and leader-follower relationships (as measured by CWT phase difference) depend on the chosen piece and take, generalized linear models were implemented to investigate the impact of empathic perspective taking (EPT) profile of the musicians on motion coordination. A total of 24 models were fitted: 1 per take/piece (thus 6 in total), per CWT measure (i.e., power and phase difference) and EPT measure (i.e., EPT group and musician’s own EPT score). We decided to investigate the effects of empathy on each take and piece (rather than nesting take under piece) since each performance might be musically unique, and therefore the effects of empathy might change between pieces and takes. Average CWT power and phase difference of the most dominant phrase level were entered in the models as response variables, whilst EPT group (i.e., LL, HH, LH, and HL) and musician’s own EPT score (i.e., singer’s EPT score and pianist’s EPT—discrete variables) were entered, once at a time, as explanatory variables. A Bonferroni correction was implemented for multiple linear models, dividing the critical *p* value level (0.05) by the number of comparisons being made, 24, corresponding to the total number of models developed for the impact of empathy on interperformer coordination. For this reason, a *p* value threshold was set at 0.002083. The model formulae related to power and EPT and musician’s own EPT score were, respectively, as follows:

$$\begin{aligned}{} & {} \mathrm{{power}} \sim \mathrm{{EPT}}\_\mathrm{{group}} \\{} & {} \mathrm{{power}} \sim \mathrm{{Pianist}}\_\mathrm{{EPT}} + \mathrm{{Singer}}\_\mathrm{{EPT}} \end{aligned}$$

The model formulae related to phase difference and EPT and musician’s own EPT score were, respectively, as follows:

$$\begin{aligned}{} & {} \mathrm{{phase}}\_\mathrm{{difference}} \sim \mathrm{{EPT}}\_\mathrm{{group}} \\{} & {} \mathrm{{phase}}\_\mathrm{{difference}} \sim \mathrm{{Pianist}}\_\mathrm{{EPT}} + \mathrm{{Singer}}\_\mathrm{{EPT}} \end{aligned}$$

**Fig. 9:**
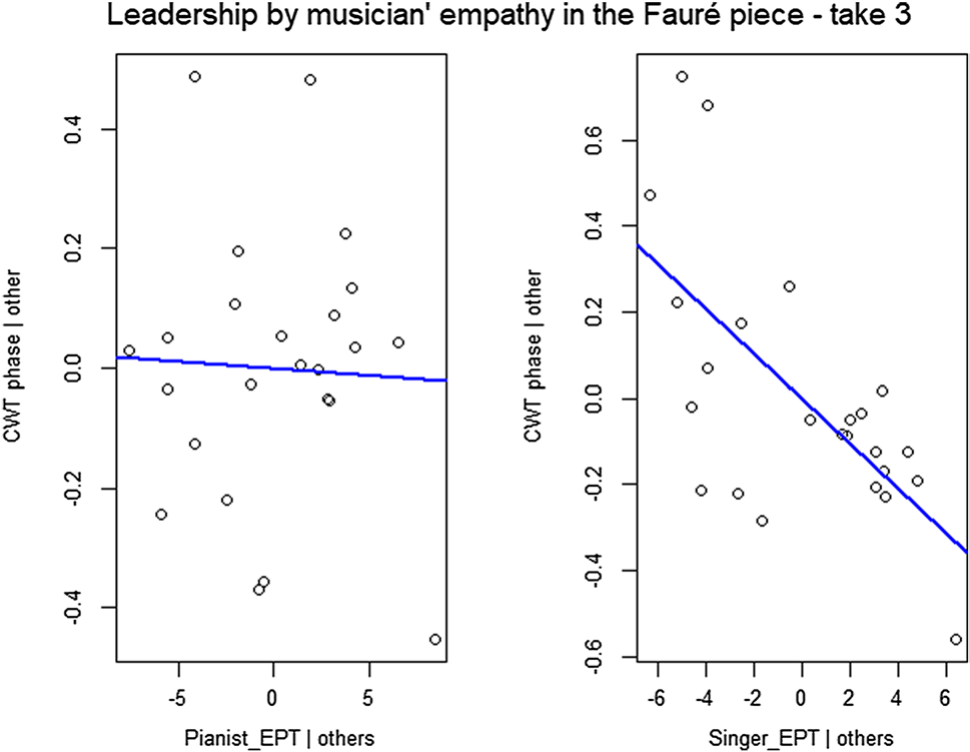
Tendency to lead/lag as measured by the CWT phase difference in the Fauré piece, take 3, plotted as a function of the musician’s own empathic score, based on a multiple linear regression model. The blue line shows the association between the explanatory variables (i.e., pianist’s EPT score on the left side plot and singer’s EPT score on right side plot) and the response variable (mean CWT phase difference), while holding the value of the other predictor variables constant (singer’s EPT and pianist’s EPT, respectively). The slope angle represents the $\beta$ coefficient. As we can see, results demonstrate that the higher the EPT score of the singer, the higher the tendency was for the singer to lead and pianist to follow; in contrast, the EPT score of the pianist did not impact the leader-follower relationships

Results demonstrate that there was no significant relationship between CWT power and the different EPT measures (i.e., EPT group and musician’s own EPT score), regardless of the piece being performed or the take number. Results also demonstrate that EPT group was not a significant predictor, regardless of the piece performed and the take number. Interestingly, singer’s EPT score had an impact on CWT phase difference of the last repeated performance of the Fauré piece: the higher the empathy score of the singer, the higher the tendency was for the singer to lead and pianist to follow $(\beta = -0.05,\; 95\%\; \mathrm{{CI}} \,[-0.08, -0.02],\; t(21) = -3.95,\; p <.001)$. In other words, in the last take of the Fauré piece, we found a significant relationship between singers’ empathy profile and the tendency to lead/follow, with a 0.05 unit increase in the tendency for the singer to lead and pianist to follow per unit of increase in singer’s EPT, as shown in Fig. 9. The model explains a substantial proportion of variance $(R^2 = 0.44,\; F(2, 21) = 8.30,\; adj.\; R^2 = 0.39)$.

This analysis of the impact of the musicians’ own EPT score on CWT power and phase difference was based on the most dominant phrase level. As explained above, results demonstrate that empathy did not predict CWT power, but did predict CWT phase difference in case of the Fauré piece, take 3. But, an analysis of the occurrences of the first three power peaks by phrase level (see Table 2 and Supplementary material analysis, Fig. 1) also highlighted that some peaks fell within less dominant phrase levels. It was therefore of interest to investigate the impact of musicians’ EPT score also based on the less strong phrase levels. For this reason, step-wise linear models were implemented (one for each piece and take) to investigate the impact of empathy on CWT power and phase difference data related to each phrase level. As shown in Table 4, empathy did not predict CWT power of any of the less dominant phrase levels, in line with the results for the most dominant phrase level. Interestingly, the singer’s EPT score did predict CWT phase difference at three bar level in the Fauré piece, take 2: the higher the singer EPT, the lower the tendency for the singer to lead and pianist to follow. These results are in contrast with the direction of the empathy impact that was found in Fauré, take 3, in which a higher singer empa

**Table 4:**
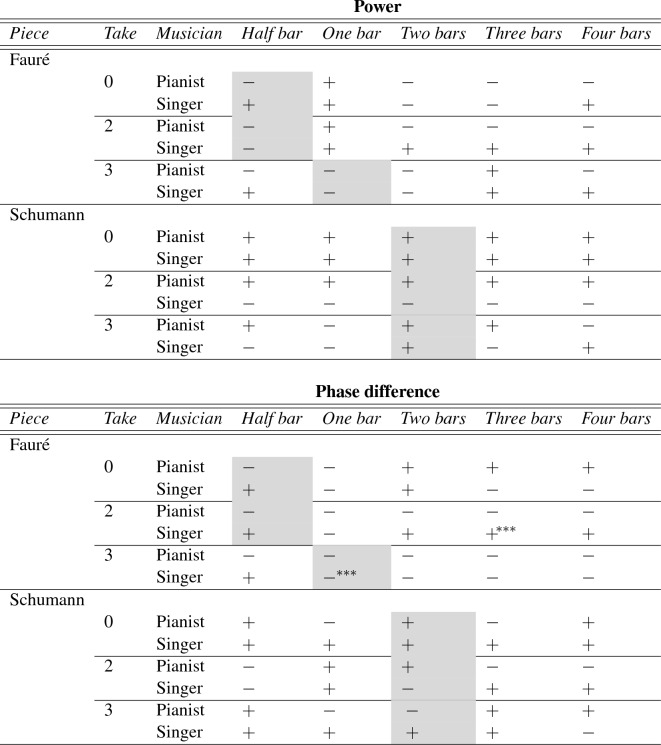
Impact of musician’s EPT score on CWT power (top) and phase difference (bottom) for each take/piece/phrase level, as measured by sign and level of significance of the $\beta$ coefficients resulting from the linear regression models implemented

$$+$$ indicates a positive relationship between empathy and CWT power/phase difference, whilst − indicates a negative relationship. Grey cells refer to the most dominant phrase level for a given piece and take. Significance code: $$^{***} 0.1\%$$

thy was associated with a higher tendency for the singer to lead and the pianist to follow.

#### Musical sections

Step 3d: Having found an impact of the singer’s empathy on the CWT phase difference in the case of the Fauré piece, take 3, a follow-up analysis was run on each take and piece as a function of the musical sections forming the piece (i.e., A, B and A’ sections for the Fauré piece and A, A’, B, A”, C, A”’ for the Schumann piece; form analysis reported in Table 1a and b, respectively). This was done to investigate whether the impact of empathy on CWT phase difference would change during the course of the piece.^4^ For this purpose, six linear nested models were fitted to predict the impact of the musical section nested under the singer’s EPT score on mean CWT phase difference. CWT phase difference data, corresponding to the most dominant phrase level identified above, were entered in the models as mean values, aggregated per sections. The phase difference model formula was as follows:

$$\begin{aligned} \mathrm{{phase}}\_\mathrm{{difference}} \sim \mathrm{{Singer}}\_\mathrm{{EPT/section}}. \end{aligned}$$

**Fig. 10:**
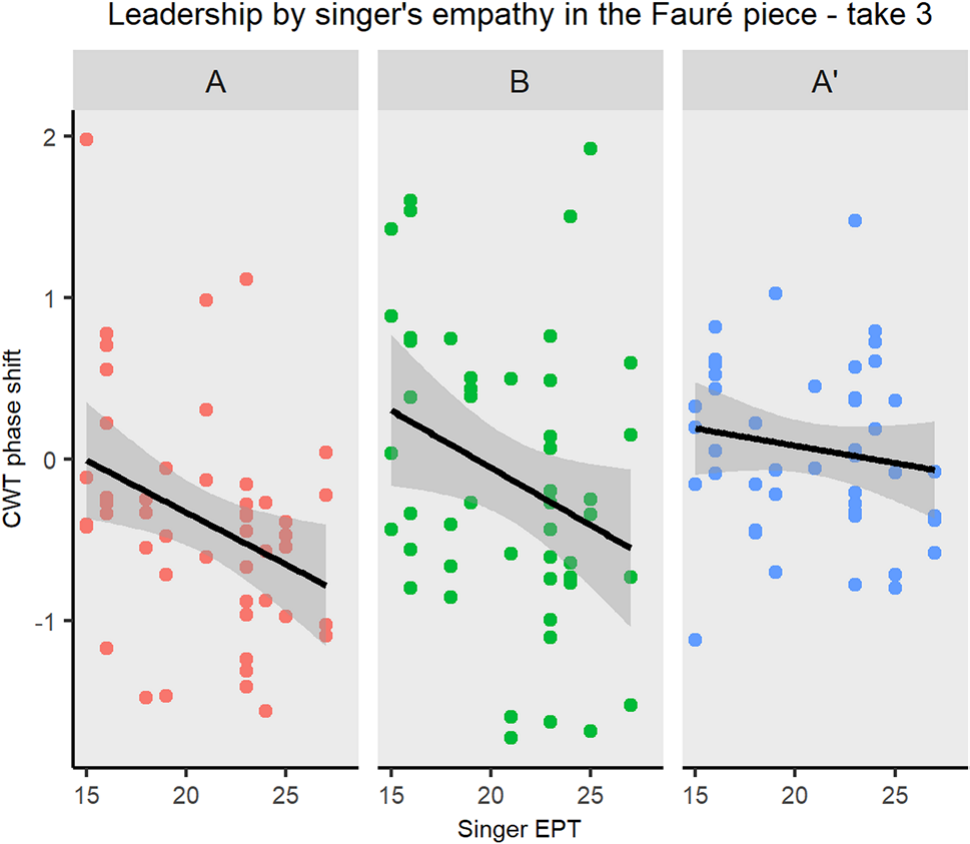
Scatter plot of the tendency to lead/lag as measured by the CWT phase difference in the three musical sections (i.e., A, B, and A’) of the Fauré piece, take 3, plotted as a function of the singer’s empathic score. The slope represents the $\beta$ coefficient. Interestingly, results demonstrate that the tendency for the singer to lead and pianist to follow in presence of more empathic singers was greater in the first section of the piece (i.e., section A), than after its repetition after the B section

As shown in Fig. 10, the direction of the influence of the singer’s EPT in take 3 of the Fauré piece was the same across the three sections: the higher the singer’s EPT, the higher the tendency for the singer to lead and pianist to follow in sections A, B and A’. A similar tendency was found above when considering the whole take. Interestingly, this tendency was greater in the first section (i.e., A) $(95\%\; \mathrm{{CI}} \,[-0.18, 0.61]$, than its repetition (i.e., A’) $(\beta = 0.02,\; 95\%\; \mathrm{{CI}} \,[8.55e-03, 0.04],\; t(138) = 3.21,\; p <.01)$. The impact of the singer’s EPT on CWT phase difference did not differ significantly between sections A and B. The model explains a moderate proportion of variance $(R^2 = 0.14,\; F(3, 138) = 7.19,\; p <.001,\; \mathrm{{adj.}}\; R^2 = 0.12)$.

Results from the models run on the remaining takes demonstrate that musicians’ own EPT did not predict CWT phase difference, regardless of the musical sections. These results are also consistent with the findings resulting from the analysis of the whole piece.

Furthermore, in light of the presence of solo moments focused on piano performance and contrasting with together sections, the analysis of the impact of empathy was conducted also as a function of the solo/together moments. To this end, six linear nested models (one per take/piece) were fitted to predict the impact of the solo/together section nested under the singer’s EPT score on mean. The CWT phase difference model formula was as follows:

$$\begin{aligned} \mathrm{{phase}}\_\mathrm{{difference}} \sim \mathrm{{Singer}}\_\mathrm{{EPT/solo}}\_\mathrm{{together}} \end{aligned}$$

**Fig. 11:**
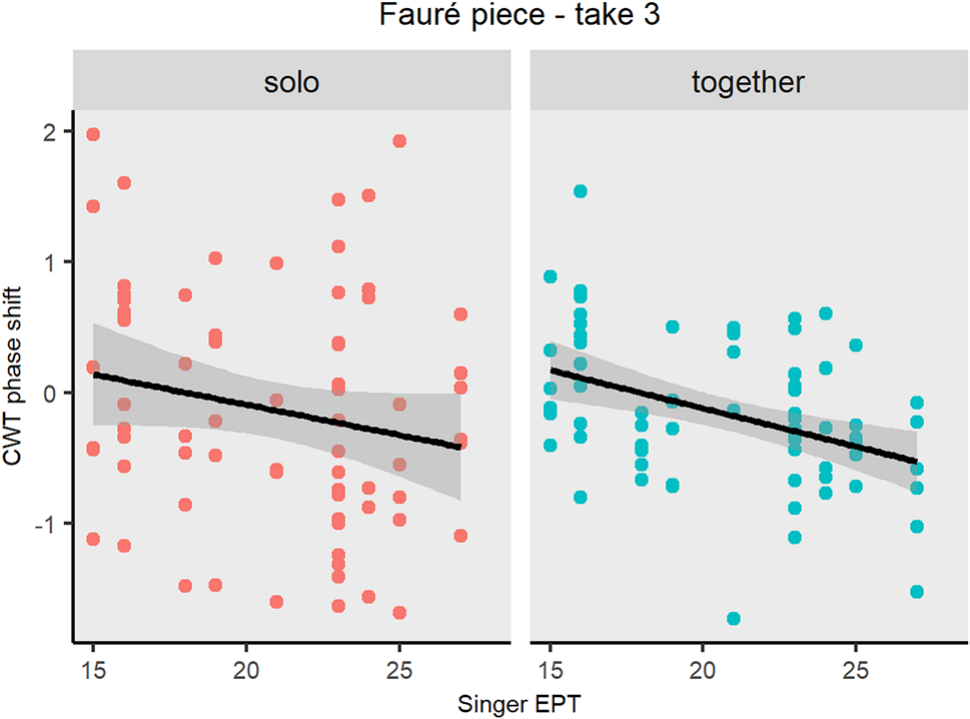
Scatter plot of the tendency to lead/lag as measured by the CWT phase difference in sections where the pianist played solo versus sections where they played together. The graph relates to the third performance of the Fauré piece and CWT phase difference is plotted as a function of the singer’s empathic score. The slope represents the $\beta$ coefficient with error bars plotted over

Results demonstrate that the impact of singer’s EPT as function of the type of performance (i.e., solo vs together) on the phase difference, i.e. the tendency to lead/lag, was consistent. In particular, for the Fauré piece, take 3, the tendency for the singer to lead and pianist to follow with more empathic singers did not change significantly between solo versus together moments, as shown in Fig. 11.

### Quantity of motion

In addition to the analysis of CWT power and phase difference of musicians’ front-head velocity trajectories, we also measured the overall quantity of motion (QoM) in performances across the 14 markers that were placed on musicians’ head and upper body. The extra four markers placed on the singer’s hip were not included in this analysis. QoM was computed as sum of 3D velocities^5^ across markers and performers per second for each duo, and then compared across pieces and repeated performances.

Two models were implemented to test the fixed effects of EPT measures (EPT group and musicians’ own EPT score, tested in separate models), take nested under piece on QoM (the response variable). Duo number was entered in both models as random effects to account for repeated measures. QoM values were aggregated by performance. The QoM model formulae were as follows:

$$\begin{aligned} \mathrm{{QoM}} \sim \mathrm{{Pianist}}\_\mathrm{{EPT}} + \mathrm{{Singer}}\_\mathrm{{EPT }}+ \mathrm{{piece/take}} \end{aligned}$$

$$\mathrm{{QoM}} \sim \mathrm{{EPT}}\_\mathrm{{group}}.$$

The analysis of changes of QoM by piece and take based on linear mixed-modelling showed that take and piece predicted QoM, which was higher in Schumann than Fauré $$(\beta = 148.7,\; 95\%\; \mathrm{{CI}} \,[140.8, 171.9]$$, $$p <.001)$$, and increased across repeated performances. For the Schumann piece, QoM was higher in take 2 $$(\beta = 85.7,\; 95\%\; \mathrm{{CI}} \,[49.4, 122],\; p <.001)$$ and take 3 $$(\beta = 87.8,\; 95\%\; \mathrm{{CI}} \,[51.5, 124.2],\; p <.001)$$ than in take 0, demonstrating that body motion increases with more practice. For the Fauré piece, QoM was also higher in take 2 $$(\beta = 75.4,\; 95\%\; \mathrm{{CI}} \,[39.1, 111.7],\; p <.001)$$ and take 3 $$(\beta = 69.2,\; 95\%\; \mathrm{{CI}} \,[32.8, 105.5],\; p <.001)$$ than in take 0, demonstrating that body motion increases with more practice also in the Fauré piece. Conversely, EPT group and musicians’ own EPT did not predict QoM, demonstrating that EPT is irrelevant for the overall energy of the performance.

## Discussion

This study focused on how head motion of piano-singing duos relates to the phrase and formal structure of the piece being performed, and musicians’ empathic perspective taking (EPT). Musicians’ head motion was investigated while performing two pieces (one from Schumann and one from Fauré) that contrasted in terms of tempo and structural features, including the clarity of the distinction between melody and accompaniment, before and after rehearsal. By applying cross-wavelet transform (CWT) analysis, we quantified the degree of similarity and phase difference in common periodicities between head velocity trajectories, as measures of the power of the interperformer coordination and the tendency to lead/lag the co-performer, respectively.

The main results can be summarised as follows:

- Peaks of interperformer coordination power predominantly corresponded to the phrase structure of the piece, demonstrating that musicians tend to jointly align their periodic head motion with a subdivision of phrase length.
- The singer’s EPT score was positively or negatively related to the singer’s tendency to lead and the pianist’s tendency to follow after a rehearsal session, depending on the take, and only for the Fauré piece (the slower and structurally simpler piece), revealing the complexity of a direct link between empathy and musicians’ head motion.

### Interperformer coordination by piece and take

Research has recently analysed how musicians’ movements during performance relate to the piece being performed (Clayton et al., 2019; Demos & Chaffin, 2017; Demos et al., 2017; Eerola et al., 2018; Thompson & Luck, 2011), by establishing a link between movements and music structure. Through the application of CWT theory to motion capture data, we provided a novel contribution to the field of music performance science, by quantifying the relationship between head motion and the phrase structure of the piece for two selections of Romantic classical repertoire. We found that a majority of the three most salient CWT power peaks fell within one of the chosen phrase levels, demonstrating, as expected (see RQ1), that interpersonal coordination power of head motion corresponds to the phrase structure of the piece.

We also found that the coordination power did not change across pieces. However, future investigations replicating this study are needed to test these results across a broad selection of pieces and verify whether advanced musicians can compensate for differences between pieces and maintain a similar strength of interactions, regardless of the piece features.

Interestingly, the averaged quantity of body motion increased with successive takes, but the strength of interaction, contrary to our expectations, did not differ across takes. This means that musicians moved more across takes but the way they periodically related to each other remained the same, which also demonstrates that quantity of body motion and periodic coordination of head motion are two different factors of interpersonal coordination. Furthermore, these results suggest that the time musicians spent together rehearsing and negotiating a common interpretation might be irrelevant for the similarity of head motion. There might still be differences in terms of sound synchronization accuracy as result of practicing together, as studies on ensemble synchronization suggest (D’Amario et al., 2018a); future investigations might analyse note-to-note synchronization in parallel to head and body motion analysis to shed more light in this respect.

Our results demonstrate that leadership, as manifested in musicians’ head motion, is shaped in large part by piece structure, regardless of musicians’ empathy (RQ 2). The overall tendency for the singer to lead and the pianist to follow that was found in the study is in line with the more traditional distribution of leader-follower roles expected in a Lied duo. But, variations in leadership intensity and direction observed between pieces, takes and musical sections within pieces do provide evidence that leadership in ensembles represents a dynamic concept rather than a more straightforward role division. This view is very much in line with studies analysing leadership in string quartets, showing complex and very well differentiated patterns of dependencies between musicians (Timmers et al., 2014).

A stronger tendency for the singer to lead and the pianist to follow in the Fauré piece than in the Schumann piece could have been induced by the pieces’ features: The piano part is more clearly presented as an accompaniment in the Fauré piece. Conversely, the pianist in the Schumann piece plays also a leader role during solo sections and when performing in unison segments of the singer’s melody. Interestingly, the strength of leadership tendency did not change within pieces during solo and together sections regardless of the piece and take, contrary to our expectations (see RQ2), whilst the interaction power was, as expected, stronger in together sections than piano solos. These results suggest that singers can play a leader role even when not required to sing notes during the short prelude and interludes of the piece. This can be understood by the role that these solo sections play: they remain part of the duo piece, implying a continuous performing and acting of both musicians, including that of singers during prolonged rests.

We observed stronger leadership in the last take of the Fauré piece after rehearsal compared with that of the first take before rehearsal (RQ3). These results corroborate evidence of a longitudinal study observing changes in the leader-follower relationships emerging spontaneously between members of a semi-professional singing quintet during five rehearsal sessions across a term of study (D’Amario et al., 2018a). Interestingly, D’Amario et al. (2018a) found evidence of a more democratic approach to leadership by the last rehearsal session, in which leadership became equally shared among the five singers, compared with earlier sessions. Conversely, we observed a stronger tendency for the singer to lead and the pianist to follow in Fauré take 3 than take 0. These apparently contrasting results could be understood in light of differences in piece features: D’Amario et al. (2018a) made use of two Bach’s chorales arranged for the study so no clear leader-follower roles were markedly induced by the pieces and leadership could emerge spontaneously during rehearsals; we, in the current study, used a Fauré piece with a clear definition of leader-follower roles. Taken together, these results suggest that leadership in ensembles evolves in the course of practicing in the direction the music piece implies. Future studies are needed to test this hypothesis across a broad selection of pieces.

Ultimately, by means of CWT phase difference, we quantified the accuracy of interperformer coordination of head motion, observing that signed asynchrony between musicians’ head motion was on average between about 400 ms and 1 s for the slower Fauré piece, and between 90 and 140 ms for the faster Schumann piece (see 3). The standard deviation of the signed asynchronies was between 500 ms and 2 s for the Fauré piece, and about 700 ms for the Schumann piece. Research analysing interperformer synchronization in Western Classical music at low-order note-to-note coordination reports typical standard deviations of signed asynchronies (and mean absolute asynchrony) between 30 and 50 ms, and mean signed asynchronies mostly close to 0 ms (Bishop & Goebl, 2015; D’Amario et al., 2018b; Goebl & Palmer, 2009; Timmers et al., 2014). Taken together, these results imply that the magnitude of interperformer note-to-note coordination tends to be smaller than that of the head motion coordination. This is not surprising in light of the fact that note synchronization in Western Classical music is highly intentional, whilst head motion is not.

### Singer’s empathy impact on coordination power and leadership

A growing body of research on empathy and music is highlighting how joint musical activities facilitate empathy (Cho, 2019; Cho & Han, 2022; Rabinowitch et al., 2012; Schellenberg et al., 2015). More recently, Novembre et al. (2019) expanded on this by demonstrating how EPT scores of untrained musicians can promote accuracy in synchronizing their actions during a joint music-making task, in which participants were required to play synchronously two different streams of musical sounds by rotating an electronic music-box.

Our study expands the work of Novembre et al. (2019), by analysing the impact of empathy in the context of naturalistic music ensemble performances, where the complexity of the music and performers’ social and musical roles in the ensemble may affect how relevant empathic abilities are, and what effect they have on ensemble interactions. We found no evidence of a relationship between empathy and interperformer coordination power of head motion trajectories, regardless of the piece performed and the take number (RQ4). Participants in our study were advanced trained musicians, with extensive experience in ensemble settings working with numerous other musicians with, presumably, different personalities. Therefore, they might have been skilled in adapting to the personality and cognitive differences that they might find amongst their co-performer(s).

Overall, these results suggest that empathy can impact interperformer synchronization skills manifested at low-order note-to-note temporal accuracy, as found in Novembre et al. (2019), but might not reflect higher-order interperformer synchronization power of head motion. Future studies might corroborate these results by analysing simultaneously the impact of EPT on interpersonal synchronization of sounds and body motion between musicians in ensembles.

We also found that the singer’s EPT impact on leadership depended on the piece being performed: empathy influenced leadership in the Fauré piece, but not in the Schumann piece (RQ4). The piano part of the Fauré piece plays a clear accompaniment role featuring a constant ternary pattern supporting the melodic line of the singer. Conversely, the piano part of the Schumann piece is less repetitive and includes instances doubling the melodic lines of the singer (see Supplementary material Fig. 2, bar 17). This more distinct distribution of leader and follower roles induced by the Fauré piece compared with the Schumann piece might have promoted the stronger tendency for the singer to lead and the pianist to follow in the Fauré piece as argued above, and might also explain differences in the empathy impact on leadership between pieces. These results suggest that the impact of empathy on leader–follower relationships might depend on the overall leadership distribution induced by the piece. Future investigations on the impact of empathy as a function of the intensity of the leader–follower roles induced by pieces, in which leadership is manipulated accordingly, might shed more light in this respect. Furthermore, the fact that in Lied-duos singers mostly perform the melodic line might explain the reason why the empathy impact on leadership was promoted by the singer and not the pianist, who plays and is expected to act as the accompaniment at stage (Frăţilă, 2021).

Interestingly, we found that the direction of the singer’s empathy impact on leadership differed between takes: the higher the singer empathy score, the higher the tendency for the singer to lead and the pianist to follow in take 3, and lower the tendency for the singer to lead and the pianist to following in take 2. These two takes took place after a rehearsal session when musicians were required to give their post-rehearsal performances as though on a concert stage. Music performances are thought to be unique in nature in terms of motivation and energy within and between musicians (Chaffin et al., 2007; Devaney, 2015); this uniqueness of music performance might explain differences in empathy impact across takes. Furthermore, when musicians were asked to identify their best take of each piece after the rehearsal session, 58% of the duos chose take 3, 35% take 2 and only 6% take 0. Musicians were very reluctant to pick their best performance as they often reported that all post-rehearsal performances were of high-level, but very distinctive quality. Musicians usually do not just propose a replication across performances, but they usually strive to provide a unique performance (Chaffin et al., 2007).

### Limitations and future directions

An important aspect to consider is that our results are related to two specific pieces from a certain repertoire, and we found some differences between pieces and consecutive takes in terms of interperformer coordination. This suggests potentially high variability across musical material and performance situations. Nevertheless, generalisability might be a difficult goal to attain, since music performance is highly situation-dependent. In addition, laboratory-based experiments such as these are highly peculiar, since they require a specific population of (semi-)professional musicians working for long periods of time. Each of our lab sessions, for example, that involved a period of setup, some rehearsal, and three repeated performances of two different pieces, lasted about 2 h in total—not to mention the time that participants spent learning the music at home before arriving for the session. An additional piece or pieces would have induced fatigue in the participants, degrading the quality of our results, and limited the number of musicians who were willing to participate in the study.

In this study, we conceptualized musical leadership as manifested in musicians’ head-motion. Such a tendency to anticipate or lag somebody else’s head motion is certainly not a comprehensive view of leadership, which is studied often in terms of social roles (Garrido & Requena, 2015; King, 2006; Lim, 2014; Page-Shipp et al., 2018) rather than body motion’s phase synchronicity. Future mixed studies might further advance our understanding of leadership in ensembles, by analysing patterns in verbal social interactions during rehearsals in combination with sound and body motion synchronization.

Furthermore, we analysed the impact of empathy in piano-singing duos. Larger ensembles with and without an assigned leader (i.e., a conductor) might provide more variability in the leader-follower relationships and the interperformer coordination, and this might also open up to an even more complex nature of the impact of empathy in ensemble playing. Notably, the range of the EPT scores of our participants (from 13 to 28) spanned roughly the top half of the possible range (from 0 to 28). Stronger effects of empathy might be undermined by insufficient heterogeneity in empathy scores. The mean EPT score of our participants is also higher than that observed by Davis (1980) among US university students. The mean EPT of our female participants, in fact, was 20.6 (SD = 4.1) and that of males was 19.2 (SD = 3.5); whilst that found by Davis (1980) was 17.96 (SD = 4.8) for the females and 16.78 (SD = 4.7) for the males. This might be a characteristics of the musician sample, since training and performance contexts can potentially promote empathy at various degrees (Sevdalis & Raab, 2014). Some self-selection issues may have affected our sample as well, since people with greater empathy might be more likely to succeed at music studies. Future studies are needed to show how the distribution of empathic profiles among high-level musicians compares to that of the general population.

Finally, we focused the analysis of interperformer coordination on musicians’ head motion, since this can signal the emotionally expressive intentions that performers aim to transmit. Musicians in ensembles often use also breathing as a visual/auditory cue to synchronize. The simultaneous analysis of body motion and breathing coordination might shed some light on the factors influencing interperformer coordination in music ensembles. In addition to head motion, pianists’ shoulders also exhibit more movements than other body parts (i.e., hip, torso, neck, elbows, wrists, middle fingers) and can reflect different expressive intentions during solo performances (Thompson & Luck, 2011). Therefore, pianists might use shoulder motion as a visual cue to facilitate coordination in ensembles, similarly to head motion.

### Conclusion

The present study provides a novel contribution to research on interpersonal coordination in music ensembles by showing that musicians’ head motion reflects the phrase structure of the piece, and the singer’s empathy score can contribute to the establishment of leader-follower relationships between musicians during piano-singing duo performances, depending on the piece being performed. These findings provide evidence, for the first time to our knowledge, of an association between empathy and musicians’ body motion in the final performances of a certain piece, by revealing how the singer’s role as leader in piano-singing duos might be emphasised or diffused by extra-musical factors like empathy. These results provide a better understanding of the mechanisms underpinning performance science and interpersonal coordination in music ensembles and during joint action activities.

## Supplementary Information

Below is the link to the electronic supplementary material.

**Supplementary material Figure 1** Automne Op. 18 N. 3 by Gabriel Fauré (pdf 570 KB)

**Supplementary material Figure 2** Die Kartenlegerin Op. 31 N. 2 by Robert Schumann (pdf 1167 KB)

Supplementary file 3 (pdf 455 KB)

## Data Availability

The datasets, including participants’ EPT scores and raw MoCap data, can be found at 10.5281/zenodo.6367015.

## Footnotes

1 Davis (1980, 1983) defined four different constructs of empathy, including Fantasy (the tendency to imagine oneself into fictional situations), Empathic Concern (the tendency to experience feelings of sympathy for others), and Personal Distress (the tendency to feel discomfort in response to distress in others) in addition to Perspective Taking defined above.

2 https://sildater.github.io/parangonada/.

3 For the purpose of this study, we used cross-wavelet power instead of wavelet coherence, which, in analogy to the concept of correlation, is normalized to give a quantity between 0 and 1, but requires further smoothing.

4 The analysis of the impact of EPT on CWT power by section was not conducted, since there was no impact of individual EPT score nor of EPT group on CWT power in the whole take in any of the pieces and takes.

5 The raw position data of the 14 markers applied to the head and upper body of each musician were smoothed using a Savitzky–Golay filter and then velocity derived, as was done for the calculation of CWT power and phase difference of the front head-marker velocity series.

## Declarations

### Public significance statements

This study demonstrates the importance of the phrase structure of the music and the empathic profile of the singer in piano-singing duos in establishing interperformer coordination power and leader-follower relationships between musicians during ensemble performances, respectively, as manifested in musicians’ body motion. These findings provide a novel contribution to research on interaction in music ensembles and performance science, by quantifying the relationships between body motion and phrase levels of the piece and revealing for the first time to our knowledge an association between body motion and empathy, in an ecologically valid situation.
