# Supplementary material for: Interperformer coordination in piano-singing duo performances: phrase structure and empathy impact
Source: Psychol Res. 2023 Apr 19;87(8):2559–82. doi: 10.1007/s00426-023-01818-8 (PMC10497663; doi:10.1007/s00426-023-01818-8)
Supplement: Supplementary file 1 — Supplementary material Figure 1 Automne Op. 18 N. 3 by Gabriel Fauré (pdf 570 KB) [file 426_2023_1818_MOESM1_ESM.pdf]

Automne

Gabriel Fauré

Op. 18 N. 3

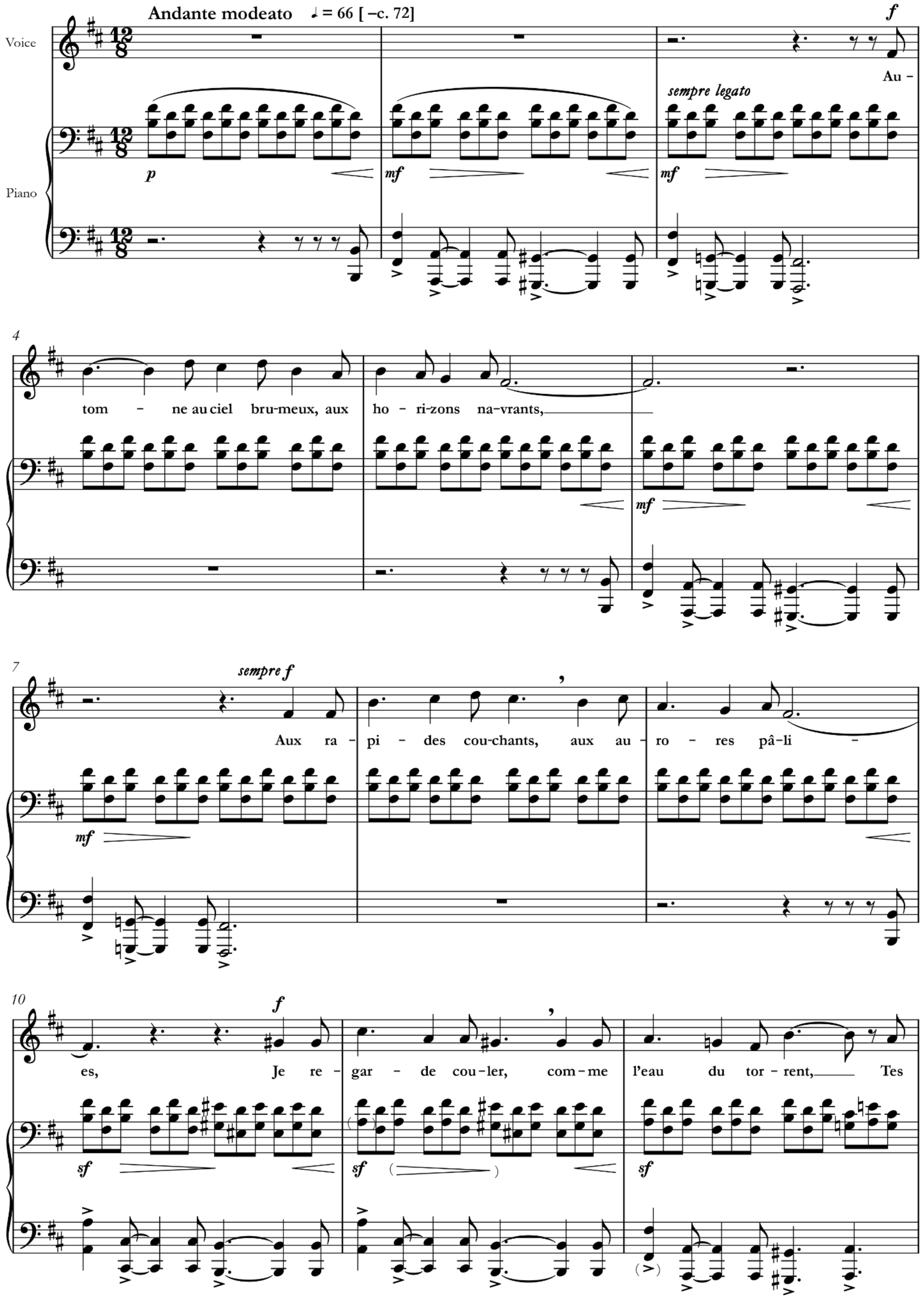

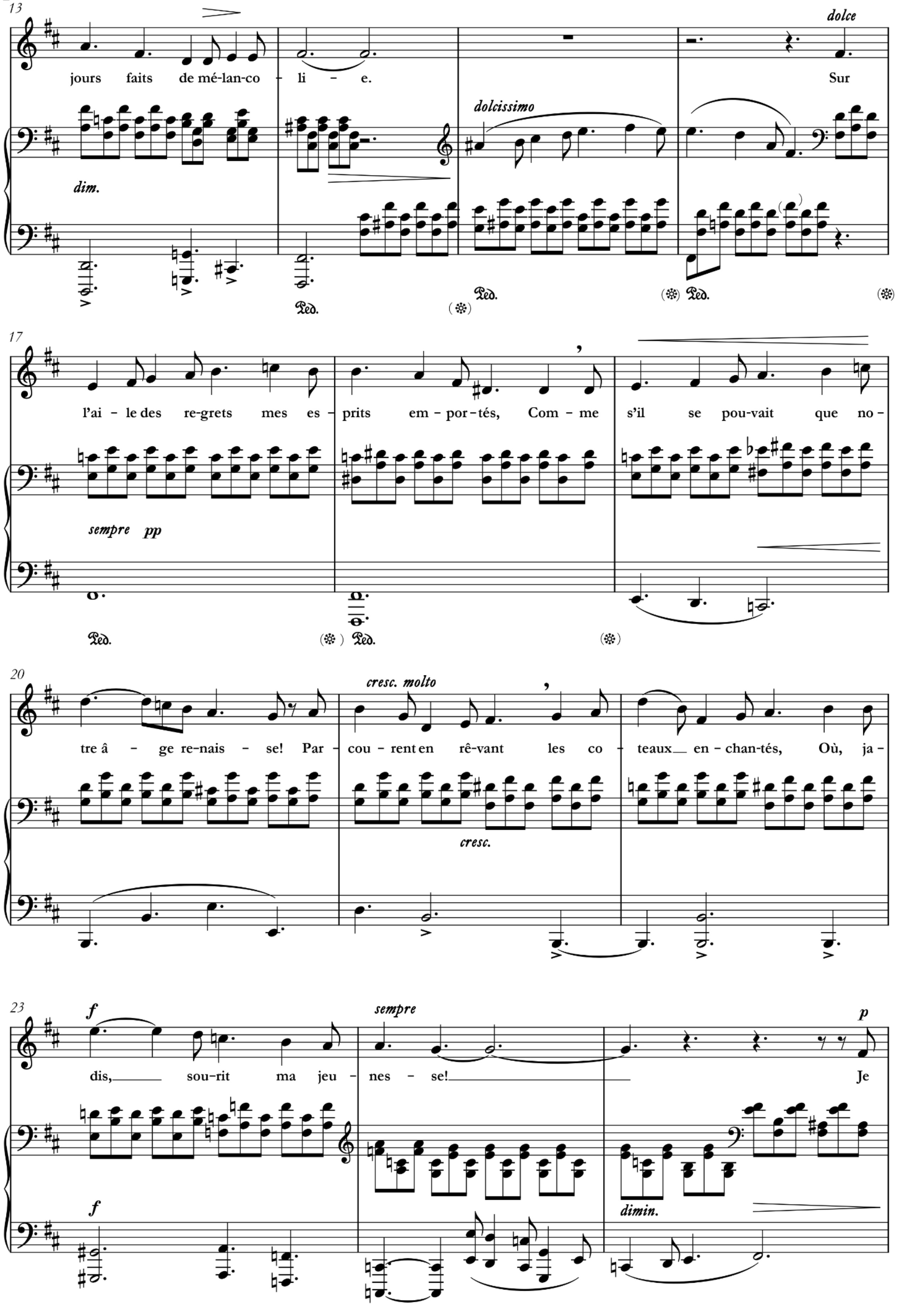
13
jours faits de mé-lan-co - li - e.
dolcissimo
dim.
dolce
Sur
17
l'ai-le des re-grets mes es - prits em - por-tés, Com - me s'il se pou-vait que no -
sempre pp
20
cresc. molto
tre â - ge re-nais - se! Par - cou - rent en rê-vant les co - teaux en - chan-tés, Où, ja -
cresc.
23
f
sempre
p
dis, sou-rit ma jeu - nes - se! Je
f
dimin.

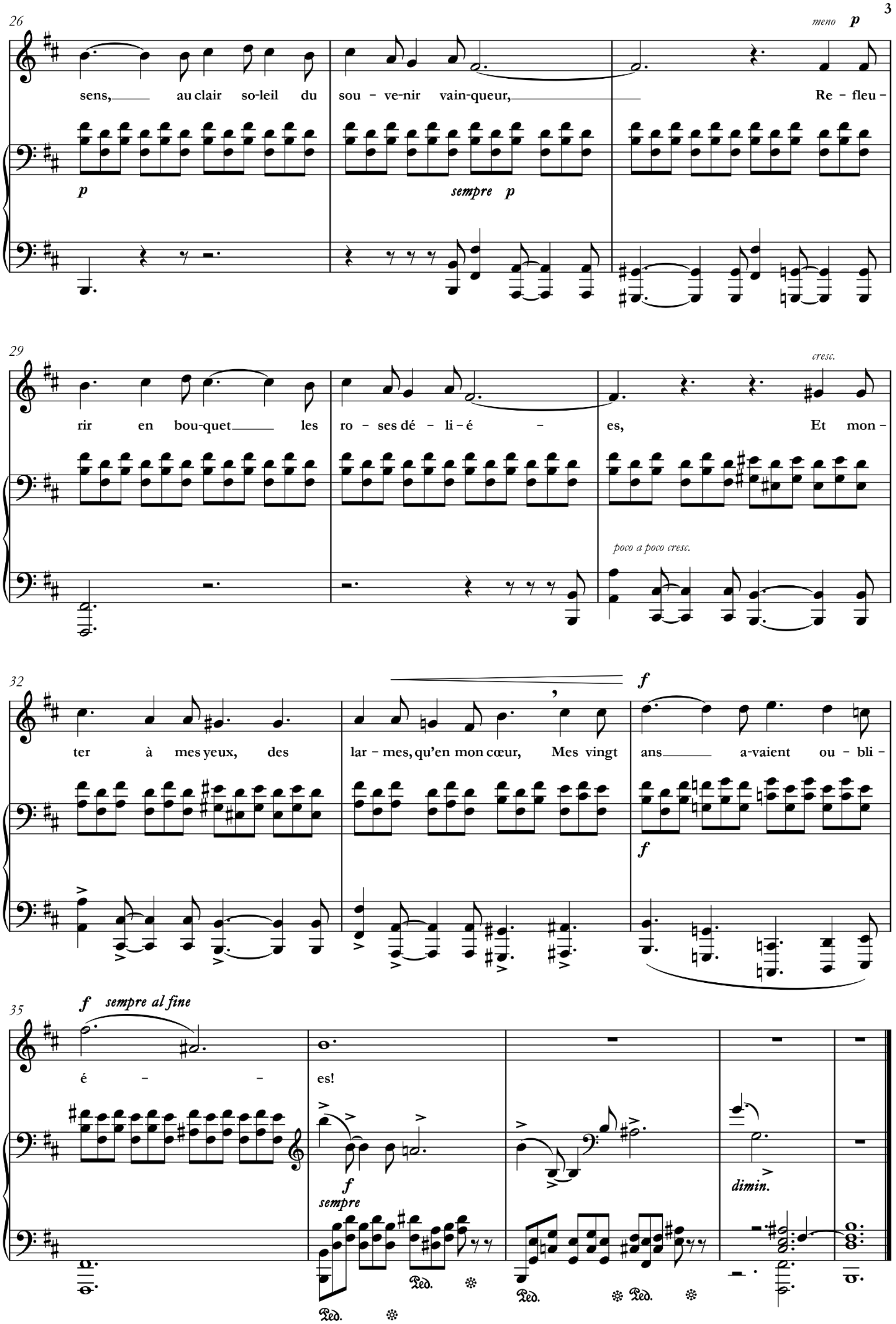
26
meno p
sens, au clair so-leil du sou-ve-nir vain-queur, Re-fleu-
p
sempre p
29
cresc.
rir en bou-quet les ro-ses dé-li-é-es, Et mon-
poco a poco cresc.
32
f
ter à mes yeux, des lar-mes, qu'en mon cœur, Mes vingt ans a-vaient ou-bli-
f
35
f sempre al fine
é-es!
f
sempre
dimin.
Ped.
