# Supplementary material for: Interperformer coordination in piano-singing duo performances: phrase structure and empathy impact
Source: Psychol Res. 2023 Apr 19;87(8):2559–82. doi: 10.1007/s00426-023-01818-8 (PMC10497663; doi:10.1007/s00426-023-01818-8)
Supplement: Supplementary file 2 — Supplementary material Figure 2 Die Kartenlegerin Op. 31 N. 2 by Robert Schumann (pdf 1167 KB) [file 426_2023_1818_MOESM2_ESM.pdf]

Die Kartenlegerin

Op. 31 N. 2

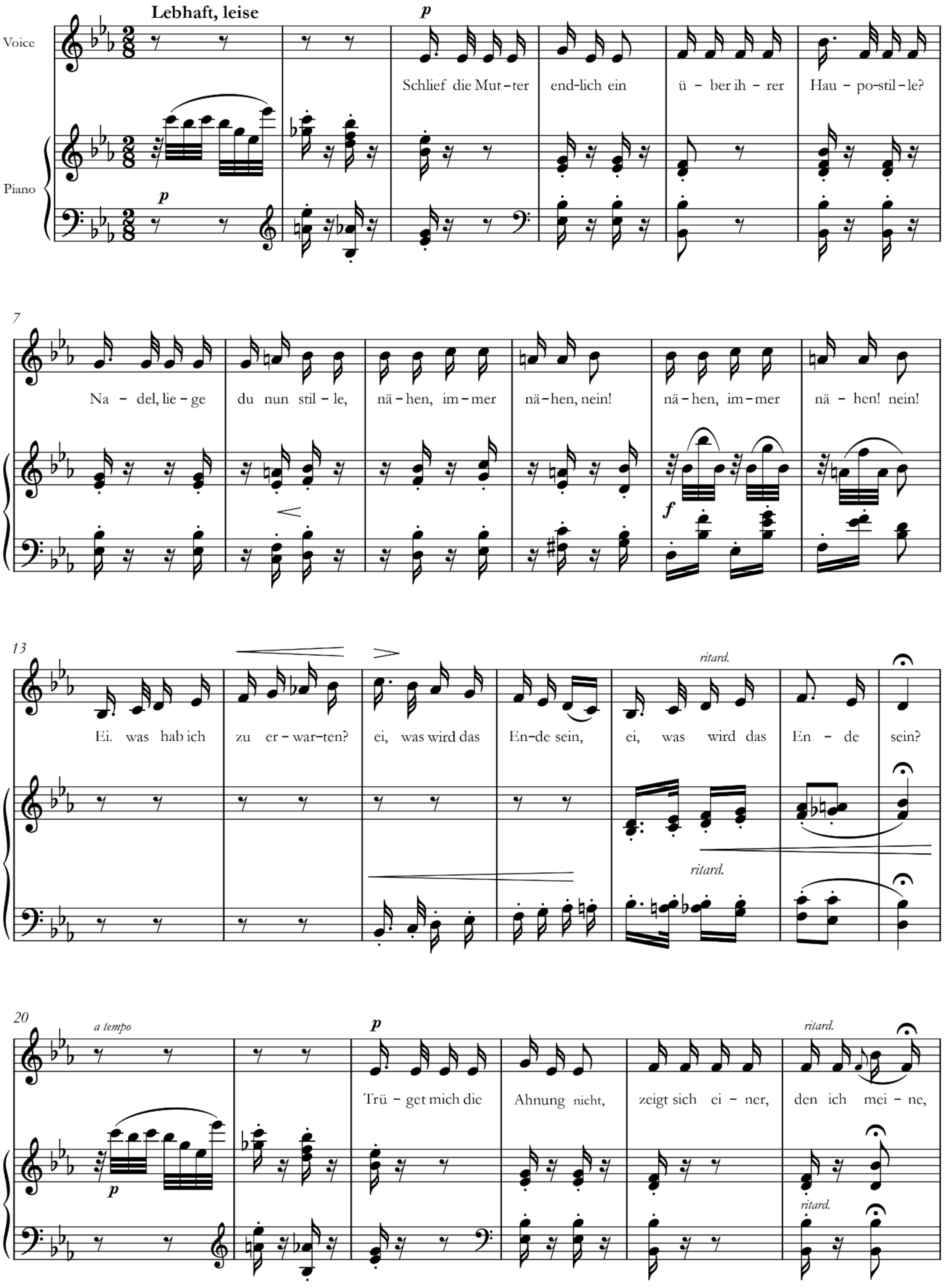

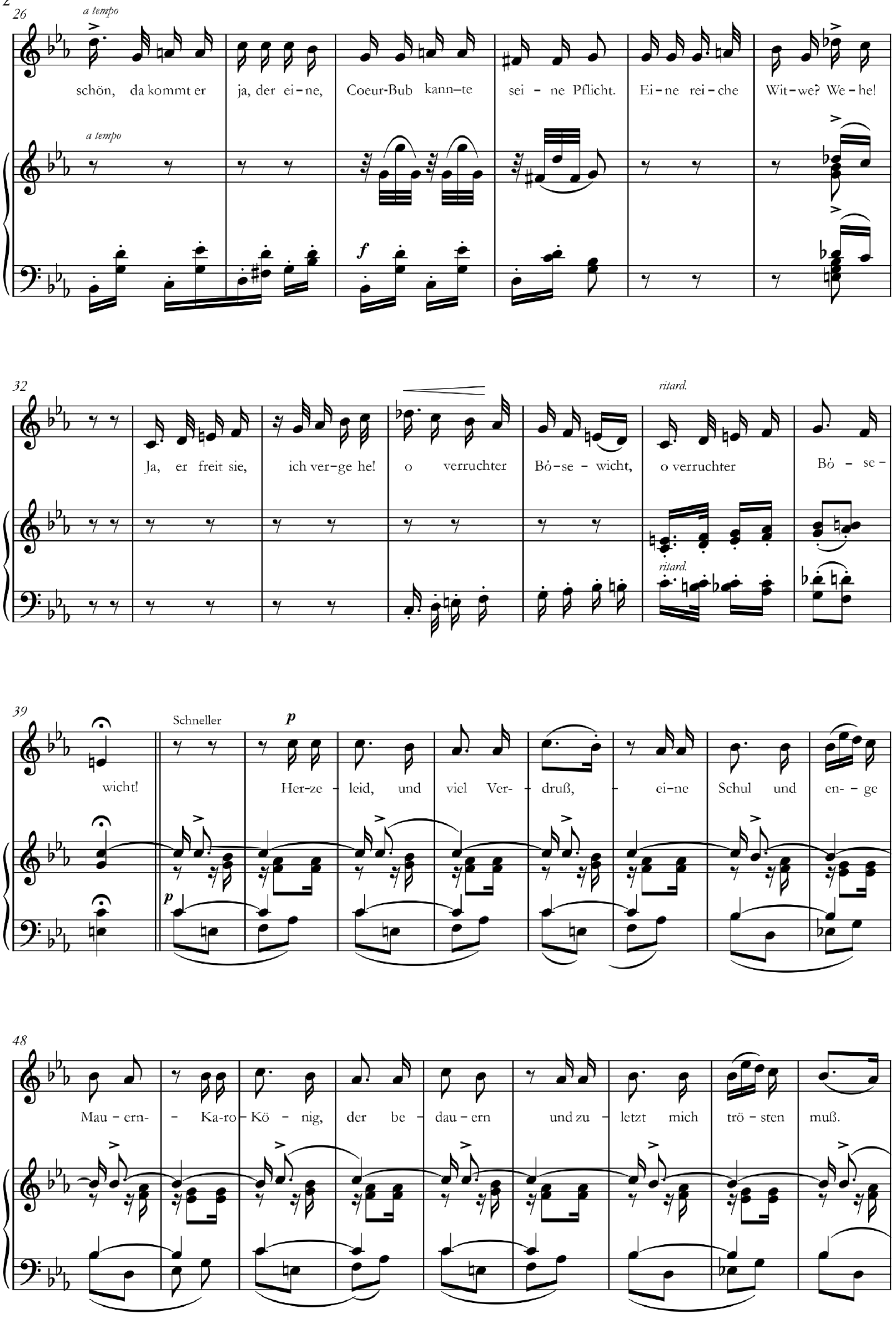
26
a tempo
schön, da kommt er ja, der ei-ne, Coeur-Bub kann-te sei - ne Pflicht. Ei-ne rei-che Wit-we? We-he!
a tempo
f
32
Ja, er freit sie, ich ver-ge he! o verruchter Bó-se - wicht, o verruchter Bó - se -
ritard.
ritard.
39
wicht!
Schneller
p
Her-ze - leid, und viel Ver - druß, - ei-ne Schul und en - ge
p
48
Mau - ern- - Ka-ro- Kö - nig, der be - dau-ern und zu - letzt mich trö - sten muß.

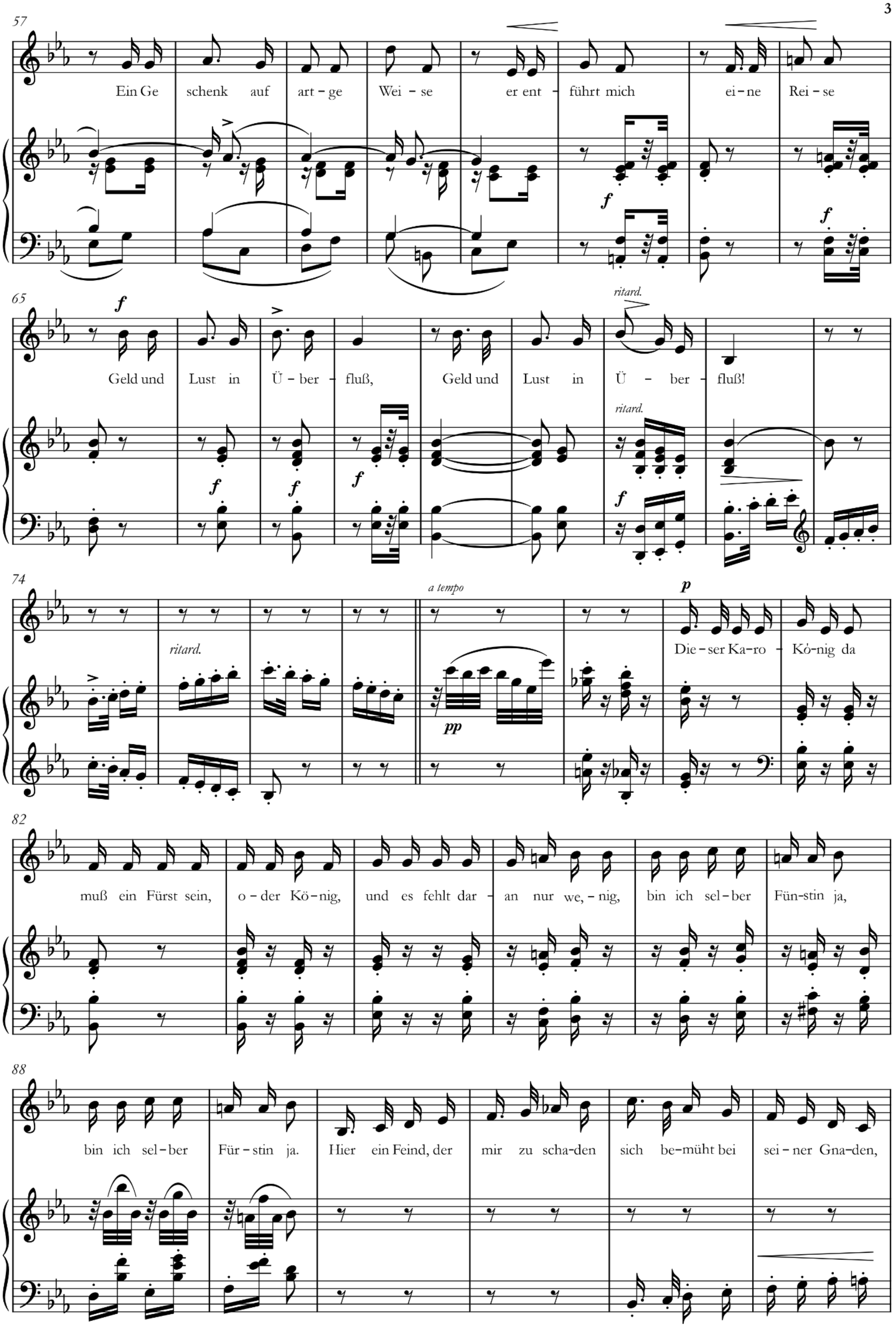
57
Ein Ge schenk auf art - ge Wei - se er ent - führt mich ei - ne Rei - se
f
f
65
f
Geld und Lust in Ü - ber - fluß, Geld und Lust in Ü - ber - fluß!
ritard.
ritard.
f
f
f
f
74
ritard.
a tempo
pp
p
Die - ser Ka - ro - Kö - nig da
82
muß ein Fürst sein, o - der Kö - nig, und es fehlt dar - an nur we -, nig, bin ich sel - ber Fün - stin ja,
88
bin ich sel - ber Für - stin ja. Hier ein Feind, der mir zu scha - den sich be - müht bei sei - ner Gna - den,

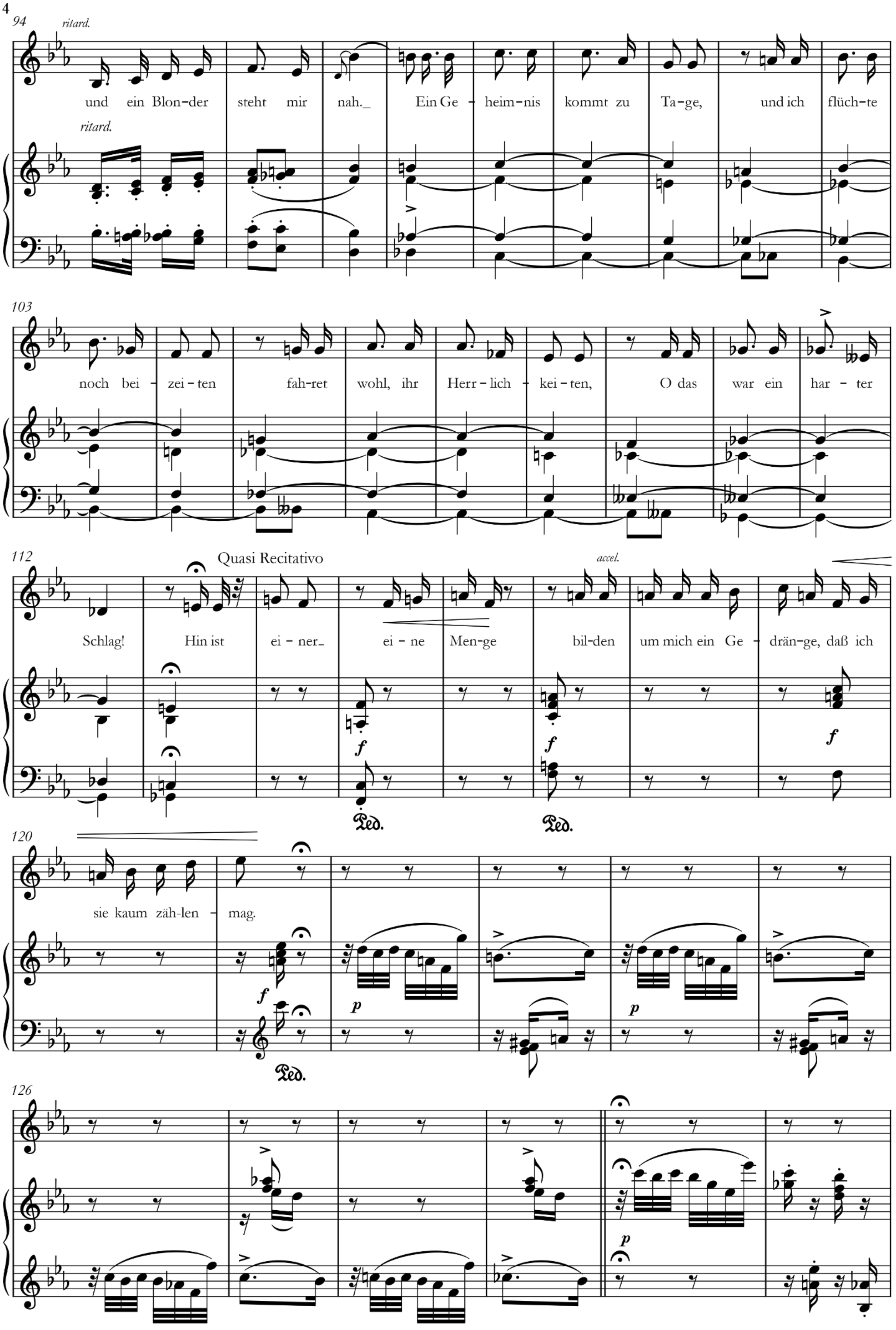
94
ritard.
und ein Blon-der steht mir nah._ Ein Ge - heim-nis kommt zu Ta-ge, und ich flüch-te
ritard.
103
noch bei - zei-ten fah-ret wohl, ihr Herr-lich - kei-ten, O das war ein har - ter
112
Quasi Recitativo
accel.
Schlag! Hin ist ei - ner_ ei - ne Men-ge bil-den um mich ein Ge - drän-ge, daß ich
f
f
f
Ped.
Ped.
120
sie kaum zäh-len - mag.
f
p
p
Ped.
126
p

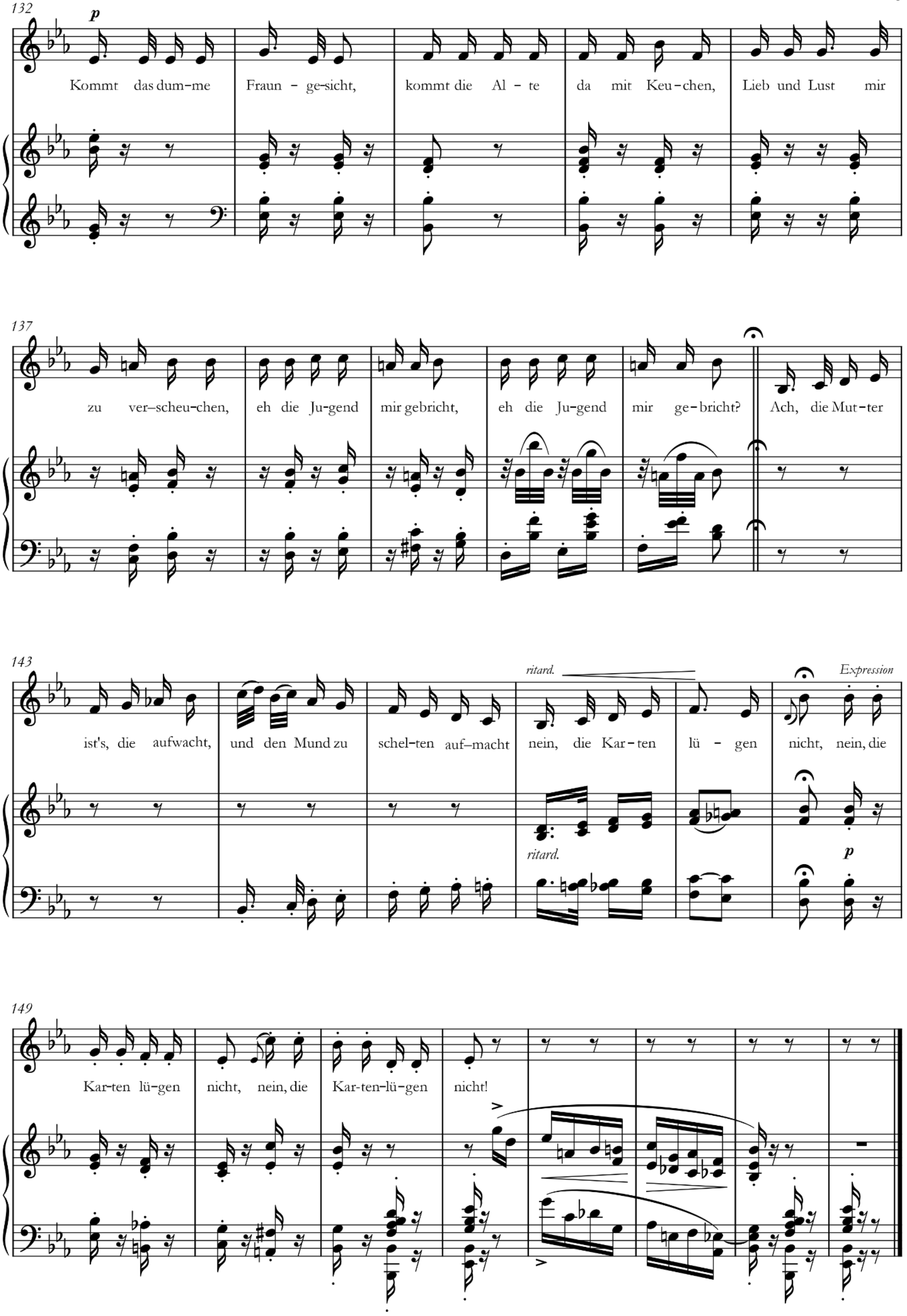
132
p
Kommt das dum-me Fraun - ge-sicht, kommt die Al - te da mit Keu-chen, Lieb und Lust mir
137
zu ver-scheu-chen, eh die Ju-gend mir gebricht, eh die Ju-gend mir ge-bricht? Ach, die Mut-ter
143
ritard.
Expression
ist's, die aufwacht, und den Mund zu schel-ten auf–macht nein, die Kar-ten lü - gen nicht, nein, die
ritard.
p
149
Kar-ten lü-gen nicht, nein, die Kar-ten-lü-gen nicht!
