# Supplementary material for: Interperformer coordination in piano-singing duo performances: phrase structure and empathy impact
Source: Psychol Res. 2023 Apr 19;87(8):2559–82. doi: 10.1007/s00426-023-01818-8 (PMC10497663; doi:10.1007/s00426-023-01818-8)
Supplement: Supplementary file 3 — (pdf 455 KB) [file 426_2023_1818_MOESM3_ESM.pdf]

Psychological Research
https://doi.org/10.1007/s00426-023-01818-8

Supplementary Material: Analysis

Robustness check: Alternative velocity data processing

In this study, CWT analysis was based on the raw velocity trajectories of the musicians' head motion, as measured using the front-head marker. However, distributions of body motion velocities were right-skewed, potentially questioning the validity of results of our statistical analyses. To reduce or remove the skewness, the log of velocity data (log + 0.1 and log + 1) was taken into account. The CWT analysis of transformed trajectories did not reveal any major changes of results, however, and thus suggested that the skewness is no serious issue.

Robustness check: Outlier sensitivity of results

The analysis of the CWT power per phrase level was based on the arithmetical mean, and we computed the grand mean of mean CWT power for each phrase level/take/piece/duo with the purpose of comparing CWT power across different phrase levels. As explained above, results from the analysis demonstrated that CWT power was strongest at two bar level for the Schumann piece, at half bar level for takes 1 and 2 of the Fauré piece, and at one bar level for Fauré, take 3.

However, the arithmetical mean is sensitive to outliers, and this might have impacted our findings. To check the robustness of the results, the analysis of the CWT power per phrase level was also conducted by: i) computing the median CWT power per phrase level, and ii) extracting the max CWT power per phrase level; medians and max power data were then aggregated for each phrase level and the related grand mean values compared. As shown in Figure 1 and Figure 2, similar relationships between the CWT power across different phrase levels were found as a function of mean, median and maximum CWT power computation. The most dominant levels were two bars for the Schumann piece, half bar for takes 1 and 2 for the Fauré piece, and one bar for Fauré, take 3, irrespective of the function used to characterize the level of CWT power across phrase levels (i.e., arithmetical mean, median or maximum power).

Furthermore, results from the analysis of musicians own EPT score on CWT phase difference in relation to Fauré, take 3, demonstrated that the singer's EPT predicted CWT phase difference, as shown in Figure 9. However, this figure also displays the presence of some outliers, related to four duos. A validity check was done by removing these outliers and re-running the model. Results from this restricted model were in line with the more comprehensive model including the outliers: singer's EPT score predicted the phase difference ($\beta = -0.03$, 95% CI[-0.05, $-5.5e-03$], $t(17) = -2.7$, $p < .05$), whilst pianist's EPT did not. The higher the empathic profile of the singer, the higher the tendency for the pianist to follow and the singer to lead in the third take of the Fauré piece.

Furthermore, the impact of empathy on CWT power was investigated by computing the grand mean CWT power related to the most dominant bar range, and no impact of empathy was found on CWT power. However, as said above, the arithmetical mean is sensitive to outliers, and these might have impacted the results. A robustness check was computed by calculating the median of the CWT power for the most dominant phrase level for each piece and take. Eventually, a model was run to investigate the impact of musicians' own EPT score on the median CWT power, for each piece and take. Similarly to the results of the impact of empathy on mean CWT power, musicians' own EPT score did not predict CWT power, regardless of piece and take. A systematic, robustness check on median CWT phase difference data was considered less necessary, since phase differences are restricted to the range from $-\pi$ to $+\pi$, and therefore are less likely to contain large outliers.

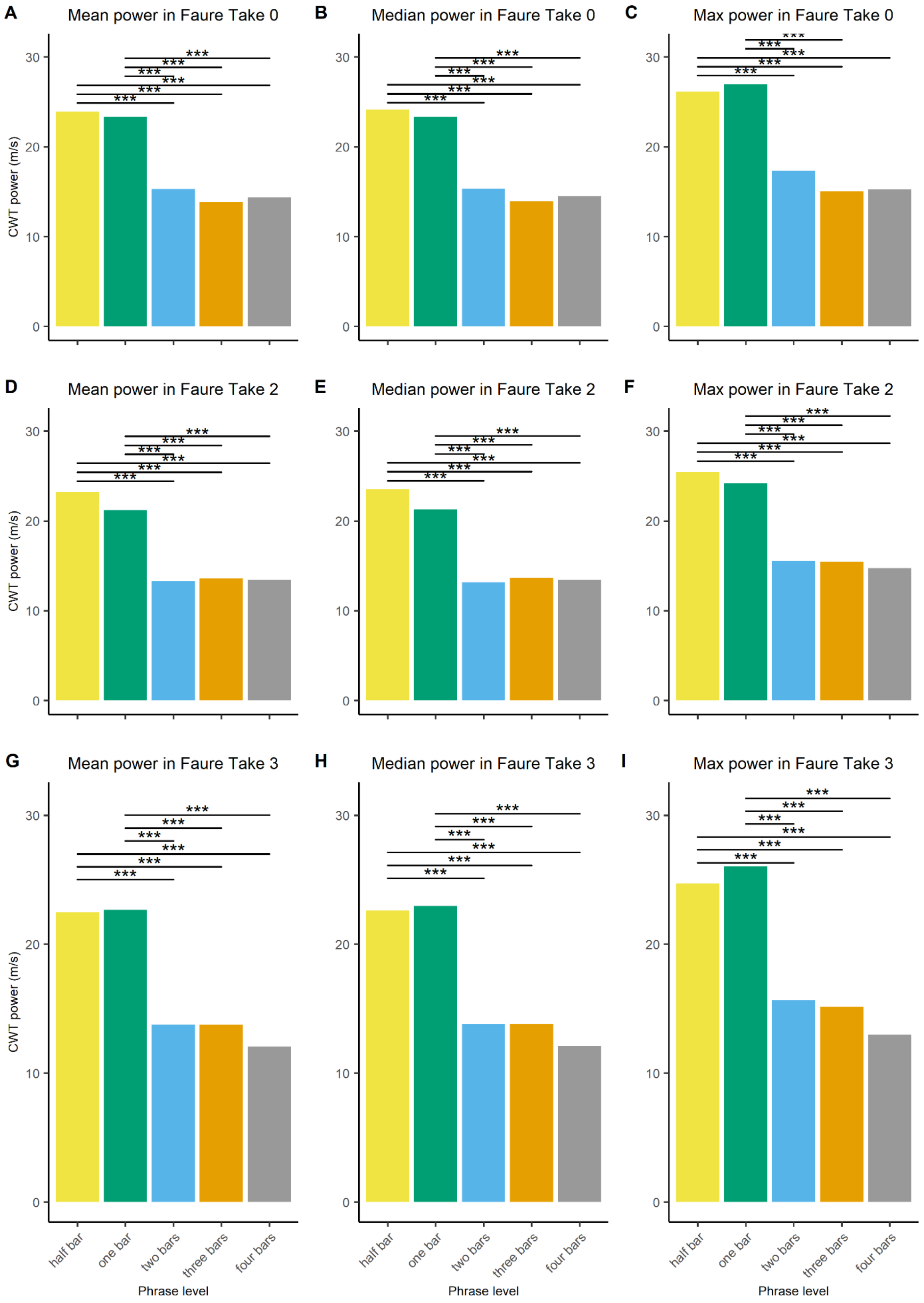

Figure 1. Grand mean characteristics of CWT power by different phrase levels (i.e., half bar, one bar, two bars, three bars, and four bars) for the three takes (i.e., take 0, take 2 and take 3) of the Fauré piece. These characteristics are based on: CWT means (left column), CWT medians (central column) and CWT maxima (right column). Horizontal lines display the significant outcomes from the Tukey test of equal means; significance codes: *** 0.1%, ** 1%,* 5%

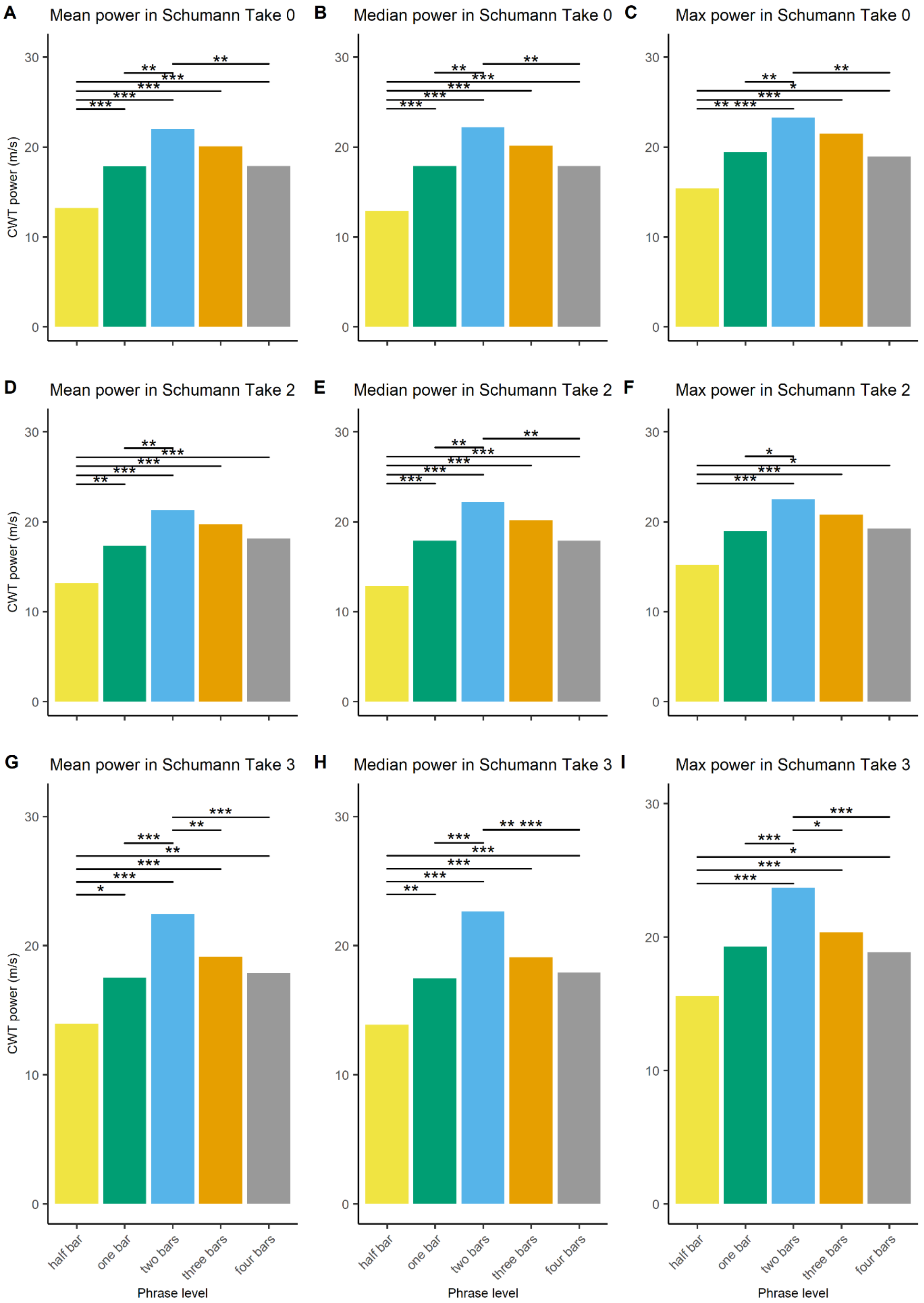

Figure 2. Grand mean characteristics of CWT power by different phrase levels (i.e., half bar, one bar, two bars, three bars, and four bars) for the three takes (i.e., take 0, take 2 and take 3) of the Schumann piece. These characteristics are based on: CWT means (left column), CWT medians (central column) and CWT max (right column). Horizontal lines display the significant outcomes from the Tukey test of equal means; significance codes: *** 0.1%, ** 1%, * 5%
